# Dynamic transcriptome profiling of Bean Common Mosaic Virus (BCMV) infection in Common Bean (*Phaseolus vulgaris* L.)

**DOI:** 10.1186/s12864-016-2976-8

**Published:** 2016-08-11

**Authors:** Kathleen Martin, Jugpreet Singh, John H. Hill, Steven A. Whitham, Steven B. Cannon

**Affiliations:** 1 Department of Plant Pathology, Kansas State University, Manhattan, KS 66506 USA; 2 ORISE Fellow, USDA-ARS, Corn Insects and Crop Genetics Research Unit, Ames, IA 50011 USA; 3 Department of Plant Pathology and Microbiology, Iowa State University, Ames, 50011 USA; 4 Department of Agronomy, Iowa State University, Ames, IA 50011 USA; 5 USDA-ARS, Corn Insects and Crop Genetics Research Unit, Crop Genome Informatics Laboratory, Iowa State University, Ames, IA 50011 USA

**Keywords:** Gene expression, Gene regulation, Host-virus interaction, Regulatory changes, Phaseolus vulgaris, Bean common mosaic virus

## Abstract

**Background:**

*Bean common mosaic virus* (BCMV) is widespread, with *Phaseolus* species as the primary host plants. Numerous BCMV strains have been identified on the basis of a panel of bean varieties that distinguish the pathogenicity types with respect to the viral strains. The molecular responses in *Phaseolus* to BCMV infection have not yet been well characterized.

**Results:**

We report the transcriptional responses of a widely susceptible variety of common bean (*Phaseolus vulgaris* L., cultivar ‘Stringless green refugee’) to two BCMV strains, in a time-course experiment. We also report the genome sequence of a previously unreported BCMV strain. The interaction with the known strain NL1-Iowa causes moderate symptoms and large transcriptional responses, and the newly identified strain (Strain 2 or S2) causes severe symptoms and moderate transcriptional responses. The transcriptional profiles of host plants infected with the two isolates are distinct, and involve numerous differences in splice forms in particular genes, and pathway specific expression patterns.

**Conclusions:**

We identified differential host transcriptome response after infection of two different strains of Bean common mosaic virus (BCMV) in common bean (*Phaseolus vulgaris* L.). Virus infection initiated a suite of changes in gene expression level and patterns in the host plants. Pathways related to defense, gene regulation, metabolic processes, photosynthesis were specifically altered after virus infection. Results presented in this study can increase the understanding of host-pathogen interactions and provide resources for further investigations of the biological mechanisms in BCMV infection and defense.

**Electronic supplementary material:**

The online version of this article (doi:10.1186/s12864-016-2976-8) contains supplementary material, which is available to authorized users.

## Background

*Bean common mosaic virus* (BCMV) is a seed borne, aphid-transmitted virus with worldwide distribution [1]. Common bean (*Phaseolus vulgaris* L.) is the main host of this virus, although it also infects other *Phaseolus* species [2, 3]. This virus can cause significant yield losses (50-100 %) in the host crop plants [4–6]. As a member of the family *Potyviridae*, it is a single-stranded, positive-sense RNA virus approximately 10 kb in length and encodes an open reading frame for a polyprotein containing 10 genes and a eleventh gene is created by ribosomal slippage in the P3 protein and is called PIPO [7–10]. Each of the 10 other proteins is cleaved out after polyprotein synthesis by one of three viral encoded proteases [7].

There are 8 pathogenicity groups in the BCMV complex, identified by the virus response on a standard set of differential bean lines [11–13]. The differential bean lines contain either a single resistance gene, or none, or combinations of stacked resistance (*R*) genes [14, 15]. Seven resistance loci have been identified: a dominant *I* locus, and recessive loci, bc-u, bc-1, bc-1^2^, bc-2, bc-2^2^, and bc-3 [11, 14, 16]. The *I* locus is associated with a cluster of *R* gene homologs belonging to the TIR-NB-LRR (Toll/interleukin- 1-nucleotide binding site-leucine rich repeat) plant immunity receptors [16]. However, the responsible gene within this locus is yet to be identified. Only one resistance locus, “bc-3”, has been identified positively (as the gene *eIF4E* [13, 17]). Although there are a number of different strains of BCMV, the sequences of these strains are closely related to one other and may recombine into new strains that are able to break resistance [13, 18, 19].

Host plants activate a number of signaling cascades to recognize different pathogens and to develop suitable defensive strategies [20–24]. Defense strategies involve differential regulation of various genes related to metabolism, signal transduction, protein modifications and other cellular functions [25] and modulation of alternate splicing patterns after the virus infection [26, 27]. Defense responses in many cases are mediated by transcription factors that control signaling pathways and other host-pathogen interactions [28, 29]. Thus, identification of various regulatory components involved in host-pathogen interactions can provide a roadmap to explore the resistance mechanisms, identify candidate genes, and development of suitable molecular markers for screening germplasm [30].

Despite the economic importance of BCMV, the molecular pathways associated with BCMV infection are not fully characterized. In order to identify host plant response upon BCMV infection, we characterized the response of common bean (*Phaseolus vulgaris* L.) to two BCMV strains on a widely susceptible common bean line, Stringless green refugee. Previous work has demonstrated that pathogenicity group I is present in Iowa and only shows symptoms on bean lines containing no known BCMV resistance genes [31]. However, during characterization of the NL1-Iowa BCMV strain, a second BCMV strain with different severity and symptoms was identified on a second susceptible cultivar, Dubbele witte. Due to the differences, this second strain was tentatively named BCMV-Strain 2 or S2. The two BCMV strains were characterized by comparing the genome sequences and the host plant responses after infection. The changes in gene expression and alternate splicing levels at two different stages of BCMV infection were evaluated using RNA-Seq data. Our results support two distinct BCMV strains, with distinct interactive profiles in the infected host plants. We describe the ways that systemic virus infection modulates various local or co-regulated defense pathways for the BCMV strains in the evaluated host plant.

## Methods

### Plant growth and virus infection

*P. vulgaris* cv. Stringless green refugee was planted in the greenhouse under ambient conditions for summer (16 h day, 8 h night). Fourteen days after planting, the first true leaves were mechanically inoculated by first dusting 600-mesh carborundum and then spreading 50 mm potassium phosphate buffer, pH 7.0, containing infectious leaf sap of BCMV NL1-I (Genbank: KM023744, [31], BCMV-S2 (Genbank: KU896809), or buffer only provided the mock inoculation.

### Strain determination of unknown virus

To further characterize the Unknown strain (BCMV-S2), a test of *P. vulgaris* cultivars obtained from the Germplasm resources information network (GRIN) was conducted to identify the strain. *P. vulgaris* cultivars Dubbele witte (PI 377736), Stringless green refugee (PI 560052), Black valentine stringless (PI 549537), Pinto UI 114 (PI 549846), Sutter pink (PI 549706), Monroe (PI 599016), Top crop (PI 554129), Imuna (PI 326420), Redland’s greenleaf B (PI 599004), Great northern UI 123 (PI 549668), Red mexican UI 34 (PI 549732), Sanilac (PI 549695), Michelite 62 (PI 549693), Great northern UI-31 (PI 549671) and Puregold wax (PI 599002) were planted in the greenhouse in individual pots under ambient summer conditions (16 h day, 8 h night). At 14 days after planting, the first true leaves were inoculated as previous. For each cultivar there was a healthy, mock inoculated plant and between 2–6 infected plants depending on seed germination. One month after inoculation, symptoms were recorded and the symptomatic leaf was selected for testing by ELISA. Leaves were ground in Indirect sample extraction buffer (provided with kit) and applied to the ELISA plate for testing with the potyvirus group test (Agdia Inc. [Elkhart, IN]) per company instructions.

### Sample harvesting, RNA isolation and sequencing

Leaf tissue was harvested from healthy and virus infected plants using three biological replicates and at several developmental stages. The first trifoliolate leaf of five plants for each replicate was collected at four days post-inoculation, then the second at six days and so on every two days until 14 days post-inoculation. Harvested tissues were immediately frozen in liquid nitrogen and were stored at −80 °C. Two leaves for each time point were tested for the presence of virus by ELISA using the general potyvirus antibody from Agdia Inc., leaving three leaves of each replicate for RNA extraction. Similarly, uninoculated leaves were used as healthy controls.

Total RNA was extracted from the infected and control leaf tissues using Qiagen RNeasy® Plant mini kit (Qiagen, Germantown, MD) as per the manufacturer’s instructions. The extracted RNA was purified using the Qiagen RNA purification kit and treated with DNaseI to remove any DNA contamination. Subsequently, RNA samples were tested for their quantity and quality using a NanoDrop spectrophotometer and Agilent 2100 Bioanalyzer. RNA was extracted from three treatment groups at two developmental stages and three biological replicates (Day 4 Healthy, Day 4 BCMV-S2, Day 4 NL1-I, Day 8 Healthy, Day 8 BCMV-S2, Day 8 NL1-I) for a total of 18 RNA sequencing libraries that were prepared using 1–3 μg of total RNA from healthy and treatment groups using TruSeq RNA Sample Preparation Kits (Illumina Inc., San Diego, CA) per the manufacturer’s protocol. Each library was barcoded and multiplexed in pools of six samples per lane. A total of three lanes were used on the Illumina HiSeq 2000. The sequencing reaction produced single-end reads of 50 bps. The sequencing was conducted at the DNA sequencing core facility, Iowa State University, Ames, IA.

### Mapping, transcript assembly, and alternate splicing analysis

The single-end raw read data obtained above was processed further, as follows. Reads were separated by sample using the barcode information. The quality of raw reads was evaluated using fastqc (http://www.bioinformatics.babraham.ac.uk/projects/fastqc/). The sequencing adapters and low quality bases at the 5 prime and 3 prime ends were removed using quality scores 20 or below in Trimmomatic [32]. Resulting reads with average quality score of 20 or below were excluded from subsequent analyses. The cleaned reads were once again tested using fastqc. The common bean genome [33] and related annotation information were downloaded from the Phytozome (http://www.phytozome.net/) and high quality reads were aligned against it using Tophat software [34]. Default settings were used for aligning the RNA-Seq reads with maximum two mismatches (−v 2). The alignment statistics were obtained using RNA-SeQC bioinformatics tool [35]. The transcript specific analysis was performed using the Tuxedo pipeline [36, 37], comprising Cufflinks2 (assembling transcripts), Cuffmerge2 (merging transcript assemblies from different samples), Cuffdiff2 (differential transcript profiling and splicing analysis). The transcripts were assembled from the mapped reads with cufflinks version 2.0.0 using the default parameters except parameter ‘-j’ (minimum depth of coverage in the intronic region, value – 0.3). The transcripts were classified based on their abundance, and lowly expressed (FPKM < 0.3) transcripts were eliminated to avoid misassembly issues due to low read count. The program Cuffmerge version 2.0.0 was used to merge the transcripts from different samples. The Cuffdiff2 tool was used to identify significant alternate splicing events (*p* value < 0.05 with FDR correction) and their fpkm values. Various splicing events were annotated using ASTALAVISTA bioinformatics software [38]. This tool can classify the extent of various alternate splicing events such as exon skipping, alternate donor, alternate acceptor, intron retention etc., and using transcript models obtained from cufflinks.

### Differential expression analysis

The mapping information was used to count the number of raw reads using HTSeq package [39]. The HTSeq software provides raw read counts for uniquely aligned reads to a single gene model and discards any reads aligning to multiple locations in the genome. The R package DESeq2 [40] was used to perform differential expression (DE) analysis between different treatments. It uses the raw read count from previous alignments to fit a generalized linear model, and estimates the differences in expression level between genes. The equation parameters are modeled using a negative binomial distribution. To call a gene differentially expressed, the DESeq2 output was filtered using two criteria. First, an adjusted p-value for multiple testing corrections with value less than 0.05 was used for DE genes. Second, genes showing less than two fold change were not considered as DE. The DE genes were categorized as up-regulated and down-regulated gene sets to perform the gene ontology (GO) analysis. The GO and functional enrichment of various biological, cellular and molecular gene classes was performed using agriGO toolkit [41]. The enrichment analysis was performed using Fisher’s exact test with a false discovery rate correction to obtain adjusted p-value for each class. A *p*-value threshold of 0.05 was used to determine significant enrichment. The KEGG (Kyoto Encyclopedia of Genes and Genomes) pathway analysis was performed using KOBAS 2.0 [42]. The transcript sequences specific to up-regulated and down-regulated genes were extracted from the common bean genome transcripts and were blasted against the KEGG pathway. The pathway enrichment was determined using Fisher’s exact test using a false discovery rate correction method [43]. A *p*-value of 0.05 or less was used as threshold to determine significantly enriched pathways. To identify the putative up-regulated or down-regulated transcription factors (TFs) in the expression data, Hidden Markov Model (HMM) motif sequences of different TFs were downloaded from Legume Transcription Factor (TF) Database [44]. This database has a collection of 61 TF families from soybean, *Lotus japonicus*, and *Medicago truncatula*. The HMM motif sequences were blasted against the DE gene sequences. The putative TFs were detected using 90 % sequence homology and e-value ≤ 10^−10^.

### *De novo* assembly of virus genomes

A virus BLAST database was built using a large collection of genomes and genes of different plant virus strains from NCBI (http://www.ncbi.nlm.nih.gov/). The unmapped raw reads from individual time points were extracted and combined separately for the NL1-I and the unknown potyvirus treatment in a single dataset. The resulting datasets were searched against the virus database to identify the virus-specific read sequences. The matched read sequences were extracted to perform a *de novo* assembly of the virus genome using default settings in the Trinity *de novo* assembly platform [45]. Viral reads were normalized to compare reads to the gene size by the following equation = ((read count single gene)/(total virus read count *kb size of gene)) *100.

## Results

### Identification of BCMV strains utilizing a *P. vulgaris* bean screen

Previously, the identification of NL1-I was conducted using a differential selection of bean cultivars. It was found that this strain successfully infected the differential bean cultivars Stringless green refugee and Dubbele witte [31]. In contrast, BCMV-S2 was tested against the same common bean panel as NL1-I [31], and was able to successfully infect the *P. vulgaris* cvs. Sutter pink, Dubbele witte, Stringless green refugee, Puregold wax and Imuna. The infection in the cvs Puregold wax and Imuna places this strain of BCMV in pathogenicity group II [14].

### BCMV infection assay during common bean development

Because BCMV-NL1-I and BCMV-S2 caused different reactions in susceptible host plants, we wanted to characterize the effects of these two isolates on host gene expression by using RNA-Seq analysis. To determine the best time points for RNA-Seq analysis, a time series analysis was conducted to establish the virus detection time in non-inoculated, trifoliolate, systematic leaves. At four days post-inoculation (dpi), virus was not detected by ELISA in the first non-inoculated trifoliolate leaves. At six dpi although no symptoms were observed, NL1-I was detected by ELISA in the same grouping of trifoliolate leaves as day 4 in two of the three replicates, and BCMV-S2 was detected in all three replicates. Both viruses were detected in the non-inoculated leaves in all replicates at the subsequent sampling times of 8, 10, 12, and 14 dpi. Plants infected by either virus became symptomatic at between 11–12 dpi. Based this time course, we used the 4 dpi (the first time point before any positives were recorded) and the 8 dpi (the day that all plants tested positive) samples for RNA-Seq analyses.

### Building virus genomes and comparative analysis

**Fig. 1:**
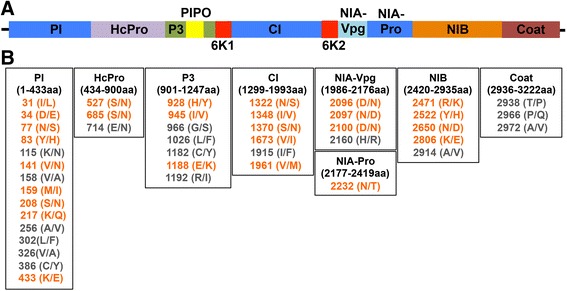
Comparative analysis of NL1-I and BCMV-S2 genomes. (**a**) The genome organization of bean common mosaic virus. (**b**) The amino acid differences between NL1-I and BCMV-S2 strains. The virus genomes from two studied virus strains were separately assembled using Trinity software [45]. The sequence span for each virus genome component is provided below the component name. The position of polymorphic sites between two virus strains is presented with the changed amino acids in brackets. The left amino acid belongs to NL1-I and right amino acid represents BCMV-S2 strain. The amino acid changes presented in grey color here, either change the conformation of the protein, binding to other proteins or function of the protein

The RNA-Seq data collected for transcriptome profiling provided the opportunity to determine the sequence of the new pathogenicity group II isolate, BCMV-S2 and confirm the identity of BCMV-NL1-I. To determine the viral sequences, we aligned the RNA-Seq reads from 8 dpi with the common bean (*P. vulgaris* L. version1) genome [33]. The proportion of unaligned reads was comparatively higher in virus-inoculated plants (12.5 %) than the healthy plants (3.6 %) at 8 dpi, which was expected due to the presence of virus transcripts along with plant genomic sequences. We utilized the unmapped reads to assemble the corresponding virus genomes for each BCMV strain (Additional file 1: Figure S1). To identify reads of virus origin, we performed a sequence similarity search of unmapped reads against different virus sequences from NCBI using blastn implemented in Basic Local Alignment Search Tool (BLAST+) [46]. Reads showing hits to various virus sequences were identified and used to build separate *de novo* assemblies for the complete genome sequences of BCMV-NL1-I (10049 bases) and BCMV-S2 (10048 bases). A search of one of the recovered and assembled viral genomes matched the NL1 genome present in NCBI (Genbank Accession AY112735), so we refer to that virus and assembly as NL1-I. A comparison of the NL1-I and the BCMV-S2 strain nucleotide sequences showed a significant number of polymorphic sites (identity = 9823/10048) between the two virus genomes (Fig. 1). This analysis confirms that BCMV-S2 is different from the NL1-I strain. There are two nucleotide changes in the 5’ UTR and one nucleotide change in the 3’ UTR, and 225 nucleotide changes in the coding regions. This resulted in a total of 45 amino acid changes between the two strains, 27 of them were changes between amino acids which had the same properties and likely to be silent mutations and 18 amino acids were changes between amino acids that had different properties (Fig. 1). There was an average of 3957 total viral reads for NL1 at 4 as compared to an average of 522,837 total NL1 reads at 8 dpi. Similarly, BCMV-S2 had an average of 32,080 total viral reads at 4 dpi and 776,986 total viral reads at 8 dpi. After normalizing the reads to account for the various gene sizes, we determined that overall the genes had equal number of reads except for P1 which contained roughly twice as many reads as any other BCMV gene (Additional file 2: Figure S4).

### Mapping statistics

To investigate the genetic mechanisms of common bean-BCMV interactions, we analyzed the RNA-Seq data to determine how alternate splicing and transcriptome profiles were altered in response to BCMV-NL1-I and BCMV-S2 at early (4 days) and late (8 days) stages of infection. The bioinformatics analysis pipeline is presented in Additional file 1: Figure S1. We obtained approximately 582 million raw sequencing reads with an average of approximately 185–200 million reads per lane, and 85–100 million reads per treatment (Table 1, Additional file 3: Table S1). Average read count for each treatment ranged from approximately 28 to 33 million. Total read count was reduced after quality filtration and barcode removal, but mostly reads were of high quality and the percentage of excluded reads was low (<1 %) (Additional file 4: Table S2). Analysis of viral reads from each treatment show much higher numbers of virus reads at day 8 than day 4 and controls – for example, 24-fold more NL1-I reads at day 8 than day 4, and 132-fold more S2 reads at day 8 than day 4 (Additional file 5: Table S3).

**Table 1:** Read statistics for healthy and BCMV inoculated treatments at 4 and 8 days after infection

	Day4 Healthy	Day4 NL1-I	Day4 BCMV-S2	Day8 Healthy	Day8 NL1-I	Day8 BCMV-S2
Total Number of Reads	100384228	85580983	95822434	100826142	101771895	97866045
Average Number of Reads	33461409	28526994	31940811	33608714	33923965	32622015
Cleaned Reads	33424094	28496111	31900317	33566283	33881828	32580889
Mapped	32016716 (95.8 %)	27359710 (96.0 %)	30558211 (95.8 %)	32359045 (96.4 %)	31295370 (92.4 %)	28489996 (87.4 %)
Unique Alignment	28953972 (86.6 %)	25106389 (88.1 %)	27610543 (86.5 %)	29033850 (86.5 %)	28563040 (84.3 %)	25955587 (80.0 %)
Multiple Alignment	3062744 (9.6 %)	2253321 (8.2 %)	2947668 (9.6 %)	3325195 (10.3 %)	2732330 (8.7 %)	2534409 (8.9 %)
Reads Unmapped	1407378 (4.2 %)	1136400 (4.0 %)	1342105 (4.2 %)	1207238 (3.6 %)	2586458 (7.6 %)	4090893 (12.5 %)
Intragenic Mapping	29974232 (93.6 %)	25976900 (94.9 %)	28844743 (94.4 %)	31105962 (96.1 %)	29702292 (94.9 %)	26984179 (94.7 %)
Exon Mapping	28665015 (89.5 %)	24837853 (90.7 %)	27654993 (90.5 %)	29549785 (91.3 %)	28357718 (90.6 %)	25632321 (90.0 %)
Intron Mapping	1309218 (4.1 %)	1139048 (4.2 %)	1189751 (3.9 %)	1556179 (4.8 %)	1344572 (4.3 %)	1351857 (4.7 %)
Intergenic Mapping	2019225 (6.3 %)	1369481 (5.0 %)	1692646 (5.5 %)	1244967 (3.8 %)	1582672 (5.0 %)	1496416 (5.2 %)
Genes Detected	21377	21179	21365	21432	21667	21400

Total number of reads represent the total count across three biological replicates, while average number of reads represent the mean value from three biological replicates. The genes were considered as expressed with minimum three reads aligned to a gene model

The quality-filtered reads were aligned against the available common bean reference genome (*P. vulgaris* v1.0). The overall mapping percentage ranged from 87.4 % to 96.4 %, with the percentage of uniquely aligned reads varying from 80.0 % to 88.1 % (Table 1). Uniquely mapped reads are particularly suitable for differential gene expression analysis [39]. Approximately 8.2 % to 10.3 % reads showed matches to multiple positions across the genome. The alignment statistics identified that most of the reads aligned to genic locations, with the highest mapping rate for exon features (89-91 %). The mapping rate to introns and intergenic regions was comparatively much lower at 4-5 % for introns and 4-6 % for intergenic regions.

The populations of viral sequences were not entirely uniform. When BCMVS2 reads are mapped against the BCMVS2 genome, there are 3 SNPs (all heterozygous, with minor allele frequency of ~10 % to 20 %); and when NL1-I reads are mapped against the NL-I genome, there are 6 SNPs (all heterozygous, with minor allele frequency of ~8 % to 45 %) (Additional files 6, 7, 8 and 9). This indicates that there was a small amount of variation in the viral populations within our samples (about 0.03 % - 0.06 % of the genomes are variant).

When BCMVS2 reads are mapped against the NL1-I genome, there are 202 SNPs (15 heterozygous); and when NL1-I reads are mapped against the BCMVS2 genome, there are 192 SNPs (4 heterozygous) (Additional files 6, 7, 8 and 9). These results indicate the degree of difference between the viral strains (approximately 2 % of the genomes are variant).

### Modulation of alternate splicing events after virus infection

**Fig. 2:**
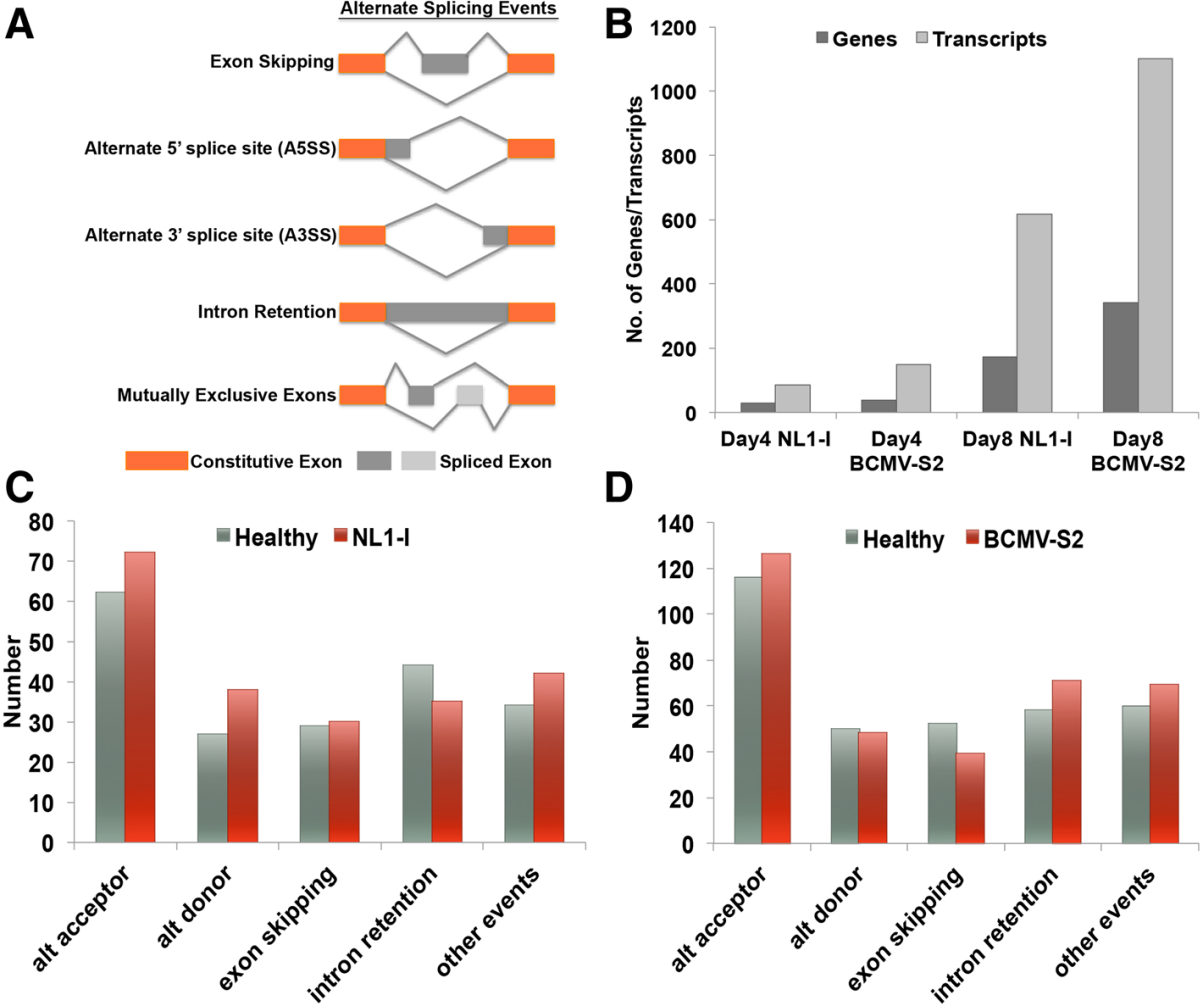
Various alternate splicing (AS) events and their distribution patterns in different virus treatments. (**a**) Representation of various AS events frequently observed in *P. vulgaris*. (**b**) Number of genes and corresponding transcripts with significant (padj < 0.05) differential splicing (AS) patterns for each virus treatments. (**c**) Different in number of different AS forms (significantly spliced transcripts) in healthy and NL1-I infected samples. (**d**) Different in number of different AS forms (significantly spliced transcripts) in healthy and BCMV-S2 infected samples. The transcripts were built using cufflinks2 and significant differential splicing events were detected using cuffdiff2 [36, 37]. Low abundance transcripts (FPKM < 0.3) were eliminated to avoid misassembly issues due to low read count. Alternate Splicing Transcriptional Landscape Visualization Tool, ASTALAVISTA [38], was used to characterize different AS events

Alternate splicing (AS) has a well-characterized role in various plant growth processes and biotic and abiotic stress conditions [27, 47–50]. We used cufflinks [36] to construct the transcripts from alignment outputs and determined various splicing patterns upon virus inoculation, using the ASTALAVISTA tool (Fig. 2a) [38]. In total, 3,194 AS events are identified in the current annotation of the *P. vulgaris* genome (Additional file 10: Figure S2, Additional file 11). Among them, alternate acceptor sites represent the largest percentage (34.5 %) of total AS events. Even though single end reads have limitations in detecting all the AS events, we were able to classify a significant number of putative splicing events in healthy and virus-inoculated plants. In our RNA-Seq datasets, the total number of predicted AS events ranged from 4,383 (Day8 BCMV-S2) to 5,245 (Day8 healthy) as shown in Additional file 12: Figure S3. Intron retention represents the dominant AS type in both healthy and virus inoculated samples (approximately 31-35 %) followed by alternate acceptor events (approximately 26-29 %), exon skipping (approximately 13–15 %), alternate donor (approximately 12–13.5 %), and other events (approximately 10–11.6 %) (Additional file 12: Figure S3). Our results mostly agree with the AS trends observed in other species [27].

To determine the statistical significance of various splicing events, we used the cuffdiff program [36, 37] to evaluate (at p-adj < 0.05) healthy and virus inoculated samples at 4 and 8 dpi. Fewer genes showed differential splicing at 4 dpi than at 8 dpi (Fig. 2b). A total of 30 and 37 genes showed differential splicing for NL1-I and BCMV-S2 at 4 dpi, which corresponds to 85 and 148 respective transcripts (Additional files 13 and 14). At 8 dpi, we found differential splicing for 172 and 342 genes for NL1-I and BCMV-S2 strains, which represent 617 and 1100 transcripts, respectively (Additional files 15 and 16). These results indicate that virus infection causes significant changes in the AS landscape of common bean leaves. Evidently, the induced virus stress over time (Day4 vs. Day8) increases the number of significant AS events (Fig. 2b). However, severe infection (Day8) is also associated with a reduction in specific AS events such as intron retention in NL1-I (Fig. 2c) and exon skipping in BCMV-S2 (Fig. 2d) treatments. A similar decrease in some AS forms has been documented under particular stress conditions [50–52]. Apparently, host machinery differentially responds to different virus strains in terms of splicing regulation.

### Regulatory changes associated with virus infection

We evaluated the coverage of our transcriptome data by considering a gene model as “expressed” if it contained or overlapped with at least three uniquely mapped reads. Approximately 80 % of the published gene models were expressed using this criterion (Additional file 17: Table S4). In order to identify the differentially expressed genes at each time point and virus treatment, we used the raw read count from the mapping output and evaluated level of gene expression with the DESeq2 statistical package [40]. A gene was considered as differentially expressed (DE) if it had a fold change of ≥ 2 (control vs. treatment) and adjusted *p*-value for multiple testing corrections less than 0.05 [43]. The DE genes were annotated using the common bean genome annotation (Phytozome.jgi.doe.gov/pz/portal.html#!info?alias = Org_Pvulgaris).

Comparison of gene expression profiles of healthy and virus treated plants at 4 dpi identified only 1 and 2 DE genes in response to NL1-I and BCMV-S2 inoculation, respectively. A single gene, Phvul.004G073400, was down-regulated under both virus treatments (log_2_ fold change was −5.5058, −4.9006 for NL1-I and BCMV-S2, respectively). This gene is the small subunit of ribulose bisphosphate carboxylase and is involved in photosynthesis. The down regulation of this photosynthetic gene in two independent virus treatments suggests that the photosynthetic pathway is highly responsive to virus infection. Another gene, Phvul.010G120600, was induced specifically in the BCMV-S2 treatment and is a NAM (“no apical meristem”) protein, involved in transcriptional regulation. Interestingly, the Phvul.005G073400 was not down-regulated at 8 dpi in either virus strain (suggesting a time point specific response), but in the S2 leaves at 8 dpi, Phvul.010G120600 continued to be induced (1.4214 at 4 dpi compared to 5.8475 at 8 dpi).

**Fig. 3:**
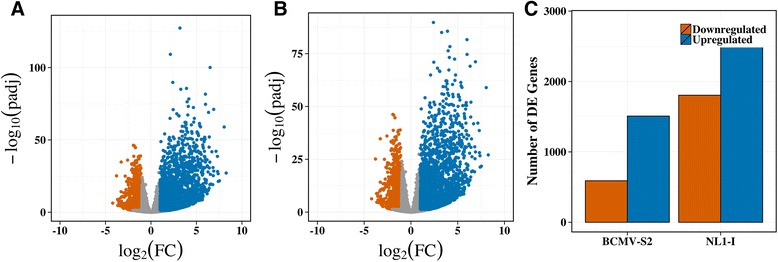
Differentially expressed (DE) transcripts (padj < 0.05) upon NL1-I and BCMV-S2 treatments. The transcripts were called as significant using adjusted p values less than 0.05 with false discovery rate (FDR) and log_2_ fold values more than 1 or less than −1. The extent and distribution of transcript expression differences were visualized using volcano plots for (**a**) NL1-I treatment, (**b**) BCMV-S2 treatments. The x-axis represent –log_10_ of adjusted p-values (FDR; [43], and y-axis represent log_2_ fold change values between control and treatment comparison. The numbers of significant DE genes were shown using bar plot (**c**), where orange color represent significantly downregulated genes and blue color represent significantly upregulated genes upon different virus treatments

**Fig. 4:**
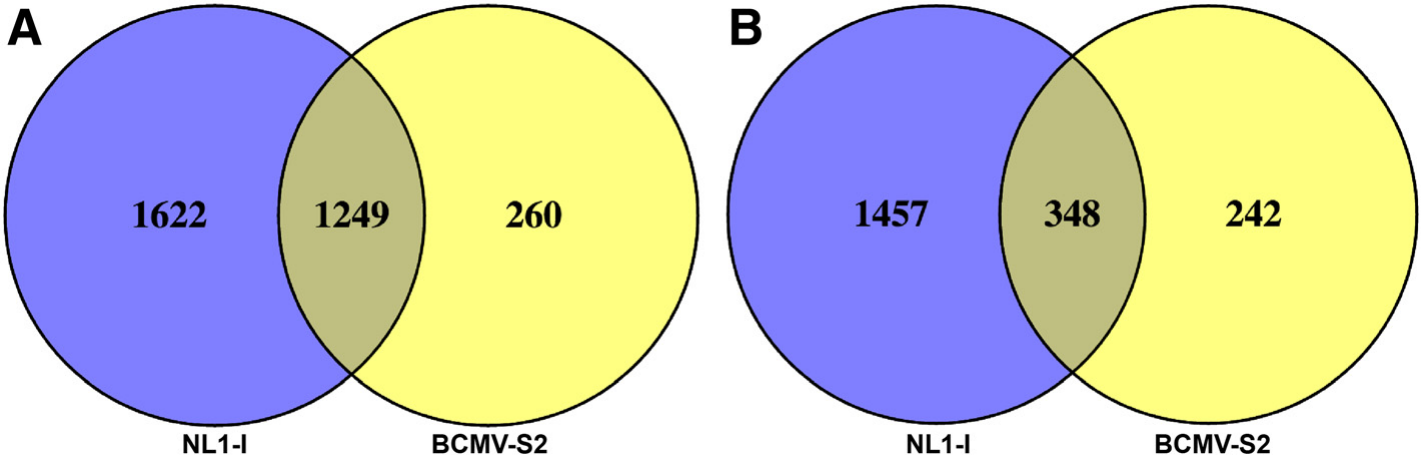
The number of DE genes uniquely expressed or shared between NL1-I and BCMV-S2 virus treatments. The induced (**a**) and repressed (**b**) genes were plotted separately for each virus treatments

At 8 dpi, we identified a total of 4676 and 2099 DE genes in response to NL1-I and BCMV-S2 treatments, respectively (Fig. 3, Additional files 18 and 19). The log_2_ fold changes were comparatively higher in the positive direction in both virus treatments (Fig. 3a, b), indicating that many genes were strongly up-regulated after virus infections. Also, a large number of genes were up-regulated in the NL1-I (2871) and BCMV-S2 treatments (1509) (Fig. 3c). The number of down-regulated genes was 1805 and 590 for NL1-I and BCMV-S2 treatments, respectively (Fig. 3c). A number of genes showed overlap between the two virus treatments (Fig. 4). To better understand the pattern of shared and unique DE genes, we plotted the induced and repressed genes separately from each virus treatment (Fig. 4a, b). A total of 1249 induced (Fig. 4a) and 348 repressed (Fig. 4b) genes were common between both the virus treatments. A total of 1622 induced and 1457 repressed genes showed NL1-I strain specific expression, while 260 induced and 242 repressed genes have a unique expression profile for BCMV-S2 virus strain. Overall, the results indicate that the NL1-I strain had a more vigorous regulatory response than the BCMV-S2 strain.

Further analysis of the top thirty induced (Table 2) and repressed genes (Table 3) revealed considerable overlap between the NL1-I and BCMV-S2 responses (27 and 21, respectively). The genes were functionally annotated using the common bean annotations (http://www.phytozome.net/). The Arabidopsis TAIR database (https://www.arabidopsis.org/) was searched to complete annotations of few unannotated common bean genes. Among the top upregulated genes, the receptor-like protein kinases (Phvul.001G043000, Phvul.004G155400, Phvul.008G109600), pathogenesis-related proteins (Phvul.002G209400, Phvul.006G196900, Phvul.006G197200), and oxidative stress related genes (Phvul.003G164600, Phvul.006G129500, Phvul.010G120300) were present (Table 2). These genes showed a common response for both the virus treatments and are frequently seen in biotic and abiotic stress conditions (reviewed in [53, 54]. Also, a WRKY DNA-binding transcription factor (Phvul.001G042200) showed a significant change in expression level (Log2 fold change 6.27 (NL1) and 4.34 (S2)) upon virus treatment. Genes in this transcription factor class are well known for their role in stress regulation (reviewed in [28, 55]. Similar analysis of the top downregulated genes identified membrane transporter classes such as transporters (vessicle, plasma membrane transporters, H+/oligopeptide, etc.) (Phvul.001G165800, Phvul.001G165900, Phvul.001G166200, Phvul.001G206700, Phvul.007G209700, Phvul.003G159200, Phvul.003G192800, Phvul.004G142000, Phvul.009G124100, Phvul.009G208200); transcription factors (Phvul.011G064900, Phvul.011G005800, Phvul.006G195500); and genes associated with photosynthetic machinery (Phvul.005G001000, Phvul.005G005000, Phvul.005G005400, Phvul.006G208300, Phvul.010G101800).

**Table 2:** List of top 30 upregulated genes (*p* < 0.05) on NL1-I and BCMV-S2 virus treatments, respectively

NL1-I	Log2 fold change	Annotation	BCMV-S2	Log2 fold change	Annotation
**Phvul.001G042200**	6.27	WRKY DNA -binding domain	Phvul.001G040600	3.97	Protein kinase domain
**Phvul.001G043000**	5.93	Leucine rich repeat N-terminal domain	Phvul.001G042100	4.27	WRKY DNA -binding domain
Phvul.001G070000	5.97	Protein tyrosine kinase	**Phvul.001G042200**	4.34	WRKY DNA -binding domain
**Phvul.001G128500**	6.82	Glycosyl hydrolases family 17	**Phvul.001G043000**	4.04	Leucine rich repeat N-terminal domain
**Phvul.002G083900**	6.70	CALCIUM-BINDING EF HAND FAMILY PROTEIN	**Phvul.001G128500**	4.50	Glycosyl hydrolases family 17
Phvul.002G180800^a^	6.47	Calcium-binding EF-hand family (0.003)	Phvul.001G192000	3.64	No apical meristem (NAM) protein
**Phvul.002G180900** ^a^	6.85	Calcium-binding EF-hand family (0.002)	Phvul.002G075200	3.62	GLUCOSYL/GLUCURONOSYL TRANSFERASES
Phvul.002G189900	6.02	VQ motif	**Phvul.002G083900**	3.96	CALCIUM-BINDING EF HAND FAMILY PROTEIN
**Phvul.002G204500**	6.08	Glucose-6-phosphate/phosphate membrane antiporter	**Phvul.002G180900** ^a^	3.76	Calcium-binding EF-hand family (0.07)
**Phvul.002G209400**	6.07	Pathogenesis-related protein Bet v I family	**Phvul.002G204500**	3.79	Glucose-6-phosphate/phosphate membrane antiporter
Phvul.003G022400	6.25	COPPER TRANSPORT PROTEIN ATOX1-RELATED	**Phvul.002G209400**	4.18	response to biotic stimulus Pathogenesis-related protein Bet v I family
**Phvul.003G164600**	5.91	Peroxidase activity, response to oxidative stress	Phvul.003G098500	3.69	Protein of unknown function (DUF679)
Phvul.003G247500	6.17	LEUCINE-RICH REPEAT-CONTAINING PROTEIN	**Phvul.003G164600**	5.57	Peroxidase activity, response to oxidative stress
Phvul.003G292900	5.98	PLAC8 family	Phvul.004G101500	3.65	Hydrolase activity, hydrolyzing O-glycosyl compounds
**Phvul.004G155400**	6.52	LEUCINE-RICH REPEAT RECEPTOR-LIKE PROTEIN KINASE	Phvul.004G142700	3.95	LEUCINE-RICH REPEAT RECEPTOR-LIKE PROTEIN KINASE
**Phvul.005G026700**	6.59	NA	**Phvul.004G155400**	3.92	LEUCINE-RICH REPEAT RECEPTOR-LIKE PROTEIN KINASE
Phvul.005G054100	6.11	Glutathione S-transferase	Phvul.005G026600^a^	4.17	Plant protein 1589 of unknown function (0.03)
Phvul.005G054200	6.59	Glutathione S-transferase	**Phvul.005G026700**	5.42	NA
**Phvul.005G171900**	6.41	WD domain, G-beta repeat	Phvul.005G038300	4.17	NA
**Phvul.006G129500**	6.24	Peroxidase activity, response to oxidative stress	Phvul.005G133400	4.03	CARBONIC ANHYDRASE (CARBONATE DEHYDRATASE)
Phvul.006G172000	6.39	NA	Phvul.005G164500	3.74	LEUCINE-RICH REPEAT RECEPTOR-LIKE PROTEIN KINASE
**Phvul.006G196900**	8.27	Pathogenesis-related protein	**Phvul.005G171900**	3.69	WD domain, G-beta repeat
**Phvul.006G197200**	8.05	Pathogenesis-related protein	Phvul.006G038100	3.62	NA
Phvul.007G040900	5.99	Peptidase	**Phvul.006G129500**	4.46	Peroxidase activity, response to oxidative stress
Phvul.008G011500	7.15	Oxidation-reduction process, C1-like domain	Phvul.006G130000	5.72	Peroxidase activity, response to oxidative stress
Phvul.008G044400	5.90	LEUCINE-RICH REPEAT RECEPTOR-LIKE PROTEIN KINASE	**Phvul.006G196900**	4.61	Pathogenesis-related protein
Phvul.008G080000	5.88	LEUCINE-RICH REPEAT RECEPTOR-LIKE PROTEIN KINASE	**Phvul.006G197200**	5.80	Pathogenesis-related protein
**Phvul.008G088700**	6.90	Tubby C 2	Phvul.007G050500	4.17	LEUCINE-RICH REPEAT RECEPTOR-LIKE PROTEIN KINASE
**Phvul.008G109600**	7.31	LEUCINE-RICH REPEAT RECEPTOR-LIKE PROTEIN KINASE	Phvul.007G211600	4.52	NA
**Phvul.008G139900**	6.90	NA	Phvul.008G088400	3.88	Tubby C 2

The bold gene names are common between the two treatments. Gene annotations were retrieved for common bean genome in Phytozome. The unannotated genes were functionally classified using TAIR (indicated by ^a^, the e-value of TAIR match is indicated in the brackets). "NA" represents no functional characterization of gene is available

**Table 3:** List of top 30 downregulated genes (*p* < 0.05) on NL1-I and BCMV-S2 virus treatments, respectively

NL1-I	Log2 fold change	Annotation	BCMV-S2	Log2 fold change	Annotation
Phvul.001G050500	−2.68	Adenine Phosphoribosyltransferase	Phvul.001G021700^a^	−2.38	Protein kinase superfamily (0.010)
**Phvul.001G091600**	−3.65	Homocitrate synthase-related	**Phvul.001G091600**	−4.08	HOMOCITRATE SYNTHASE-RELATED
**Phvul.001G138800** ^a^	−3.31	dsRNA-binding protein 3 (DRB3) (0.007)	Phvul.001G091700^a^	−3.24	ARABIDOPSIS HEAVY METAL ATPASE 8 (0.029)
**Phvul.001G165900**	−2.75	ABC-2 type transporter	**Phvul.001G138800** ^a^	−2.68	dsRNA-binding protein 3 (DRB3) (0.007)
Phvul.001G206500^a^	−2.79	Hexokinase 3 (0.006)	Phvul.001G151900	−2.87	Terpene synthase family, metal binding domain
Phvul.001G239100^a^	−3.00	Encodes a microRNA that targets several TIR1/AFB family members (0.003)	Phvul.001G165800	−3.38	ABC transporter
Phvul.002G153600	−2.80	Hydroxymethylglutaryl-CoA synthase	**Phvul.001G165900**	−3.05	ABC-2 type transporter
Phvul.002G193300	−3.00	Oxidoreductase, 20G-Fe(II) oxygenase family protein	Phvul.001G166200	−2.42	ABC-2 type transporter
**Phvul.002G216900**	−3.24	Squalene Monooxygenase	Phvul.001G206700	−2.53	ABC transporter
**Phvul.002G259700** ^a^	−3.15	Transposable element gene (0.092)	Phvul.001G251300	−2.34	TREHALOSE-6-PHOSPHATE SYNTHASE
Phvul.002G268800	−3.79	EamA-like transporter family	Phvul.001G263900	−2.36	Protein of unknown function (DUF1264)
**Phvul.002G297200** ^a^	−3.35	RING/U-box superfamily protein (0.055)	Phvul.002G059500	−2.33	LEUCINE-RICH REPEAT RECEPTOR-LIKE PROTEIN KINASE
**Phvul.002G297300** ^a^	−3.53	Mediator complex subunit Med23 (0.075)	Phvul.002G150200	−2.74	Drug transmembrane transporter activity
Phvul.003G159200	−2.91	Plasma membrane H + −transporting ATPase	**Phvul.002G216900**	−2.48	SQUALENE MONOOXYGENASE
Phvul.003G192800	−2.81	H+/oligopeptide symporter	Phvul.002G227500^a^	−2.69	Encodessl dehydroquinate-shikimate dehydrogenase enzyme (2e-08)
Phvul.003G218900	−3.27	Protease inhibitor/seed storage/LTP family	**Phvul.002G259700** ^a^	−2.32	Transposable element gene (0.092)
Phvul.003G248200	−2.98	Alpha/Beta Hydrolase Fold-Containing Protein	Phvul.002G294200	−2.80	AAA-FAMILY ATPASE
**Phvul.003G278200**	−3.03	Glucose dehydrogenase/choline dehydrogenase/mandelonitrile Lyase	**Phvul.002G297200** ^a^	−2.55	RING/U-box superfamily protein (0.055)
Phvul.004G142000	−3.17	Synaptic vesicle and related transporters	**Phvul.002G297300** ^a^	−3.13	Mediator complex subunit Med23 (0.075)
**Phvul.005G001000**	−3.24	Cellulose synthase	Phvul.003G060100	−2.92	Dehydrogenases with different specificities
Phvul.005G005000	−2.83	ATP binding	Phvul.003G082300	−2.36	OXIDOREDUCTASE, 2OG-FE(II) OXYGENASE FAMILY PROTEIN
**Phvul.005G005400**	−2.69	UDP-glucoronosyl and UDP-glucosyl transferase	**Phvul.003G278200**	−2.59	Glucose dehydrogenase/choline dehydrogenase/mandelonitrile lyase
**Phvul.005G032500**	−3.68	Dirigent-like protein	**Phvul.005G001000**	−2.49	Cellulose synthase
**Phvul.005G032600**	−3.28	Dirigent-like protein	**Phvul.005G005400**	−2.47	UDP-glucoronosyl and UDP-glucosyl transferase
Phvul.005G069400	−3.09	NA	**Phvul.005G032500**	−3.09	Dirigent-like protein
Phvul.005G145700	−2.72	DVL family	**Phvul.005G032600**	−2.73	Dirigent-like protein
**Phvul.005G159600**	−3.25	GTP-binding protein	**Phvul.005G159600**	−2.94	GTP-binding protein
Phvul.005G166200	−4.20	Phosphorelay signal transduction system	Phvul.006G195500	−2.54	Helix-loop-helix DNA-binding domain, transcription regulator activity
Phvul.005G170300	−3.09	Aquaporin (major intrinsic protein family)	**Phvul.007G209700**	−3.09	ABC transporter
Phvul.006G034000	−2.71	NAD dependent epimerase/dehydratase family	Phvul.007G275800	−2.38	Hemopexin

The gene names in bold are common between the two treatments. Gene annotations were retrieved for common bean genome in Phytozome. The unannotated genes were functionally classified using TAIR (indicated by ^a^, the e-value of TAIR match is indicated in the brackets). "NA" represents no functional characterization of gene is available

### Response of plant pathways to BCMV infection

**Fig. 5:**
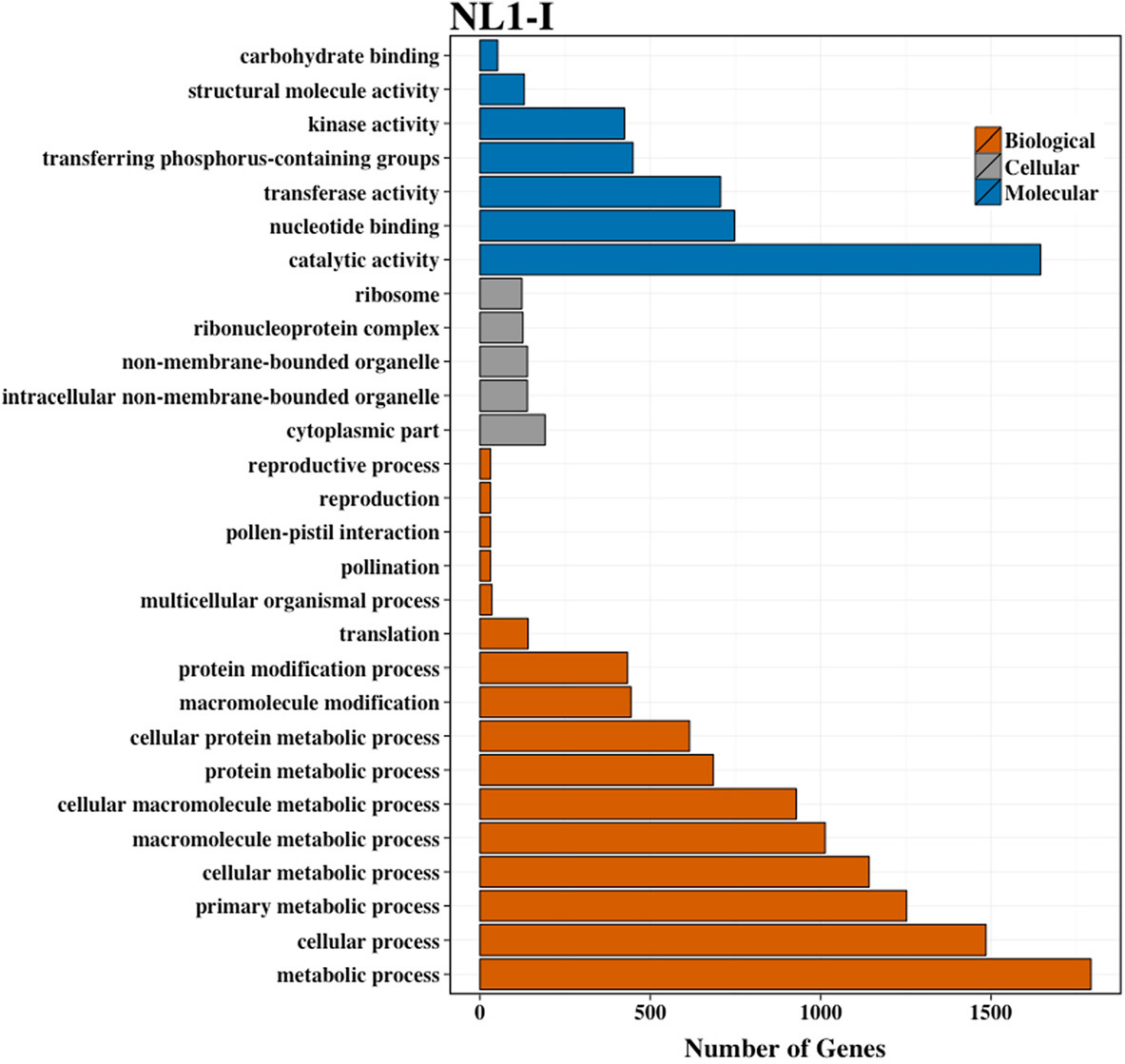
Significantly enriched GO slim categories after NL1-I virus treatment. Significant DE genes (padj < 0.05, log_2_ fold −1,1) identified after NL1-I treatment were used to perform GO enrichment analysis. The number of genes within each category is represented on x-axis. The significant overrepresented GO classes (false discovery rate corrected *p*-value < 0.05) were identified using agriGO analysis tool [41] *Phaseolus vulgaris* version1 genome annotations

**Fig. 6:**
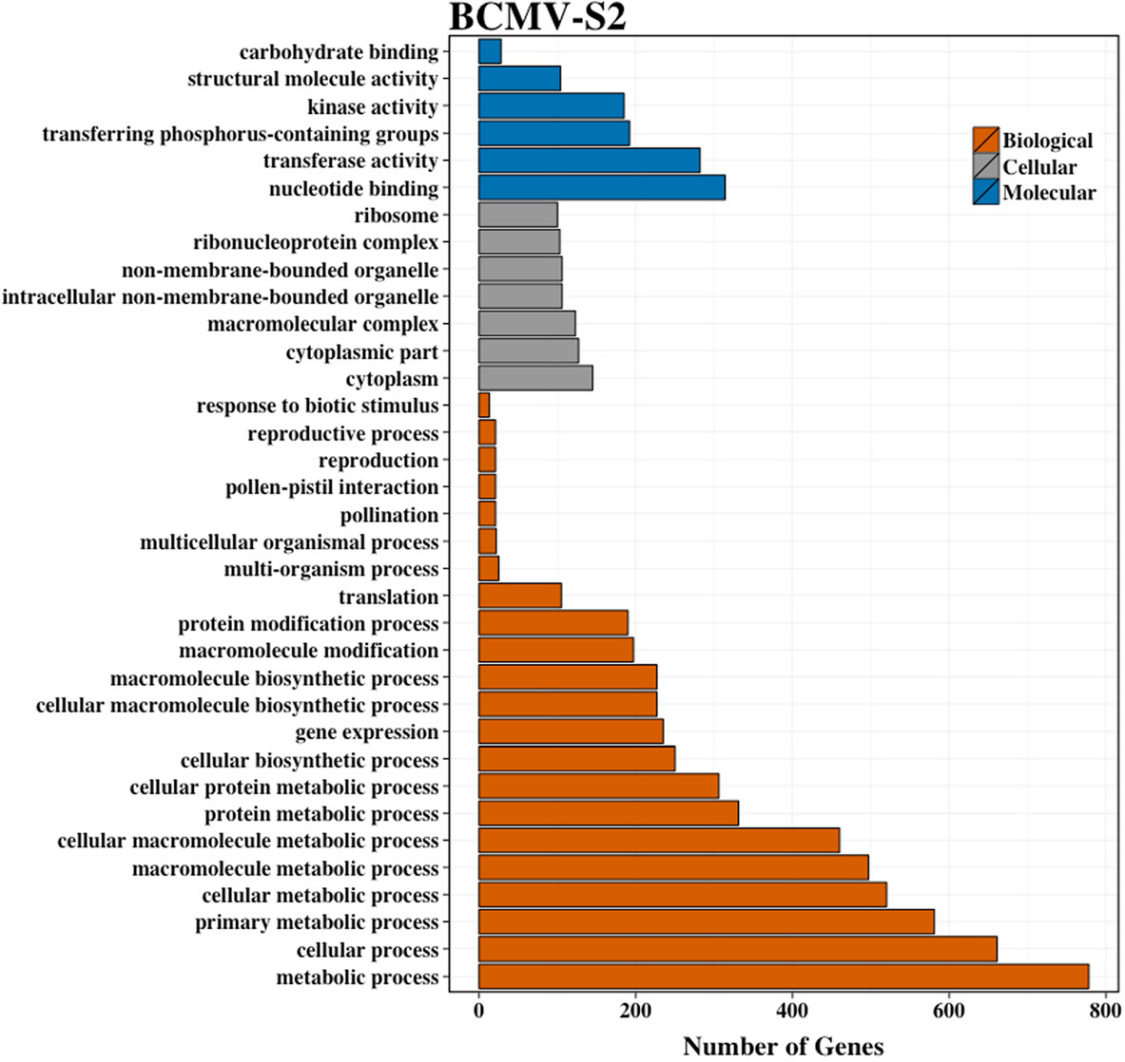
Significantly enriched GO slim categories after BCMV-S2 virus treatment. Significant DE genes (padj < 0.05, log_2_ fold −1,1) identified after BCMV-S2 treatment were used to perform GO enrichment analysis. The number of genes within each category is represented on x-axis. The significant overrepresented GO classes (false discovery rate corrected *p*-value < 0.05) were identified using agriGO analysis tool [41] using *Phaseolus vulgaris* version1 genome annotations

To discern information about pathways affected by virus infection, significantly overrepresented (padj < 0.05) gene ontology (GO) terms associated with DE genes were analyzed at 8 dpi for each virus treatment. The analysis was performed with respect to all genes present in the common bean genome. Various overrepresented GO terms associated with biological, molecular and cellular processes were identified for both NL1-I (Fig. 5, Additional file 20) and BCMV-S2 treatments (Fig. 6, Additional file 21). Similar pathways representing a wide range of functions were activated for each virus treatments The significantly induced overrepresented pathways correspond to cell death (GO:0008219), cell communication (GO:0007154), cellular metabolic process (GO:0044237), cytoplasm (GO:0005737), gene expression (GO:0010467), kinase activity (GO:0016301), metabolic processes (GO:0008152), nucleotide binding (GO:0000166), pollination (GO:0009856), protein metabolic process (GO:0019538), protein modification process (GO:0006464), receptor activity (GO:0004872), reproductive process (GO:0022414), response to stress (GO:0006950), ribonucleoprotein complex (GO:0030529), ribosome (GO:0005840), transferase activity (GO:0016740), and translation (GO:0006412). Comparatively fewer pathways were downregulated in response to virus infection. Various repressed pathways belong to photosynthesis (GO:0015979), catalytic activity (GO:0003824), transferase activity (GO:0016740), and carbohydrate metabolic processes (GO:0005975).

We also performed a pathway analysis using KEGG (Kyoto Encyclopedia of Genes and Genomes) to understand the relation between gene expression pattern and associated pathways for each virus treatment. Different datasets were created for upregulated genes, downregulated, unique, and shared DE genes for each virus treatment. The complete KEGG pathway analysis is presented in Table 4. The KEGG analysis using all DE genes indicated that various pathways with functional classes related to processes involving ribosomes, ribosome biogenesis, photosynthesis, plant-pathogen interaction, and metabolism were perturbed by virus infection. These pathways were commonly enriched in both the virus treatments. However, uniquely expressed genes in the two treatments showed enrichment of different pathways. Photosynthesis antenna proteins, pentose phosphate pathway, protein processing in endoplasmic reticulum, N-glycan biosynthesis, stilbenoid, diarylheptanoid and gingerol biosynthesis, protein export, biosynthesis of secondary metabolites, and nitrogen metabolism pathways were significantly enriched (*p* < 0.05) using unique DE genes for the NL1-I treatment. In contrast, ribosome, ribosome biogenesis, and diterpenoid pathways were enriched (*p* < 0.05) using uniquely expressed genes under the BCMV-S2 treatment.

**Table 4:** KEGG pathway analysis using DE genes on NL1-I and BCMV-S2 virus treatments

Treatment	Dataset	Pathway	DE Genes	*P*-Value
NL1-I	Unique	Plant-pathogen interaction	20	0.049
		Photosynthesis - antenna proteins	12	0.001
		Pentose phosphate pathway	16	0.006
		Protein processing in endoplasmic reticulum	39	0.014
		N-Glycan biosynthesis	12	0.020
		Protein export	12	0.028
		Biosynthesis of secondary metabolites	149	0.040
BCMV-S2	Unique	Nitrogen metabolism	9	0.049
		Ribosome	23	0.000
		Ribosome biogenesis in eukaryotes	7	0.001
		Diterpenoid biosynthesis	3	0.007
NL1-I, BCMV-S2	Common	Ribosome	79	0.000
		Ribosome biogenesis in eukaryotes	22	0.000
		Plant-pathogen interaction	20	0.006
NL1-I	Downregulated	**Photosynthesis - antenna proteins**	14	0.000
		**Sesquiterpenoid and triterpenoid biosynthesis**	9	0.001
		Biosynthesis of secondary metabolites	119	0.001
		**Starch and sucrose metabolism**	36	0.002
		Metabolic pathways	207	0.003
		Carbon fixation in photosynthetic organisms	15	0.003
		Glyoxylate and dicarboxylate metabolism	13	0.006
		Pentose phosphate pathway	12	0.008
BCMV-S2	Downregulated	**Photosynthesis - antenna proteins**	4	0.011
		**Starch and sucrose metabolism**	14	0.014
		Linoleic acid metabolism	4	0.019
		Valine, leucine and isoleucine biosynthesis	3	0.035
		Valine, leucine and isoleucine degradation	4	0.050
		**Sesquiterpenoid and triterpenoid biosynthesis**	3	0.050
NL1-I	Upregulated	**Ribosome**	111	0.000
		Protein processing in endoplasmic reticulum	46	0.000
		**Plant-pathogen interaction**	41	0.000
		**Glutathione metabolism**	22	0.000
		**Ribosome biogenesis in eukaryotes**	22	0.000
		Phenylalanine metabolism	25	0.002
		N-Glycan biosynthesis	12	0.003
		Phenylpropanoid biosynthesis	33	0.004
		Protein export	12	0.005
BCMV-S2	Upregulated	**Ribosome**	102	0.000
		**Ribosome biogenesis in eukaryotes**	28	0.000
		**Plant-pathogen interaction**	17	0.004
		**Glutathione metabolism**	11	0.004

The analysis was performed using KOBAS v2.0 [42] by splitting the DE genes dataset into unique, shared, upregulated and downregulated genes for studied virus treatments. The pathways in bold letters are significant at adjusted *p*-value < 0.05

To discern the identity of various induced and repressed pathways upon virus infection, we used the upregulated and downregulated genes separately for KEGG analysis (Table 4). Pathways specific to photosynthesis antenna proteins, sesquiterpenoid and triterpenoid biosynthesis, and starch and sucrose metabolism were commonly repressed (*p* < 0.05) under both virus treatments. In contrast, certain downregulated gene specific pathways were unique to each virus treatment. For example, biosynthesis of secondary metabolites, metabolic pathways, carbon fixation in photosynthetic organisms, glyoxylate and dicarboxylate metabolism, and pentose phosphate pathway were unique to NL1-I treatment, while linoleic acid metabolism, alpha-Linolenic acid metabolism, valine, leucine and isoleucine biosynthesis, diterpenoid biosynthesis, and valine, leucine and isoleucine degradation were downregulated (*p* < 0.05) in the BCMV-S2 treatment. Similar analysis was conducted using the upregulated genes. The results indicated that pathways related to ribosome, ribosome biogenesis, plant pathogen interaction, and glutathione metabolism were shared between two virus treatments and were significantly upregulated (*p* < 0.05). Certain pathways such as protein processing in endoplasmic reticulum, phenylalanine metabolism, N-glycan biosynthesis, phenylpropanoid biosynthesis, and protein export were uniquely upregulated after the NL1-I treatment, while no unique pathway was observed for the BCMV-S2 treatment.

### Clustering patterns illustrated specific modules related to overrepresented GO terms

**Fig. 7:**
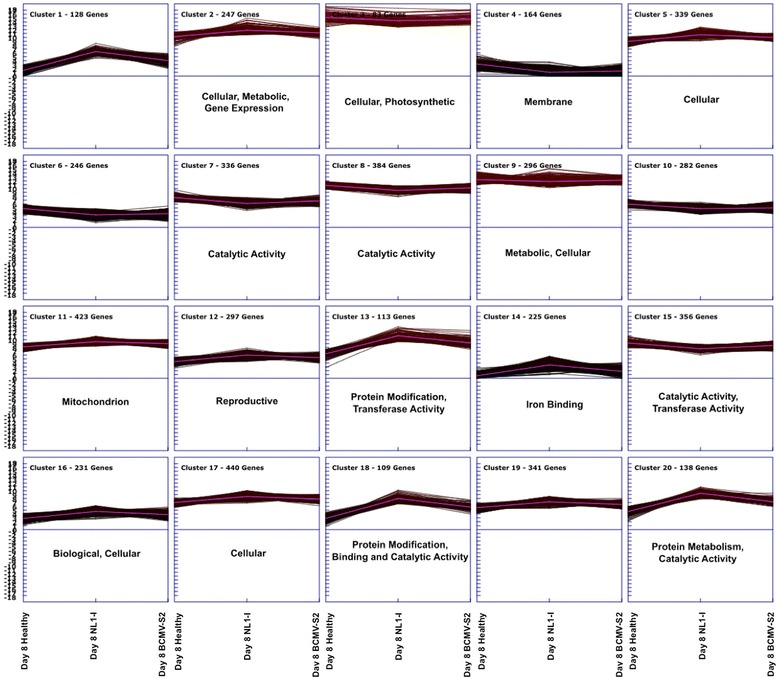
Expression modules associated with DE genes 8 days after NL1-I and BCMV-S2 virus infections. Cluster analysis was performed with k-means (*n* = 20) using Genesis bioinformatics software [56], (http://genome.tugraz.at). Normalized gene expression values were used and averaged across biological replicates for clustering analysis. The x-axis represents the healthy, NL1-I and BCMV-S2 treatments, respectively. The y-axis represents the normalized expressed values obtained from read counts for each models. Enriched gene ontology (GO) terms for various expression modules are indicated

Similar expression patterns between different genes often illustrate functional correlation between them. To identify various expression modules related to specific pathways, the DE genes were clustered with k-means clustering using Genesis software [56] http://genome.tugraz.at). The normalized expression values from the DE genes in each virus treatment were combined to obtain twenty distinct clusters (Fig. 7, Additional file 22). Also, the GO enrichment analysis (as described earlier) was performed using genes within each module to correlate the gene expression patterns with specific functional categories. The number of co-expressed genes varies for different clusters. For instance, the largest clusters represent cellular processes (cluster 17) with 8.5 % of total DE genes (*n* = 440). This is followed by mitochondrion processes (cluster 11) having 8.2 % of total genes (*n* = 423). Similarly, clusters 3 and 18 have the smallest sizes (89 and 109 genes, respectively) and represent enriched pathways in cellular, photosynthetic, protein modification, binding and catalytic activity. Furthermore, clear patterns are evident in up regulation (cluster1, cluster 13, cluster18) and down regulation (cluster4, cluster6, cluster7).

The GO enrichment analysis, using genes in individual clusters, showed that a number of clusters belong to one specific functional category. For example, specific functional classes such as membrane (cluster4, *n* = 164), catalytic activity (cluster7 and 8, *n* = 336 and 384 respectively), mitochondrion (cluster11, *n* = 423), reproductive (cluster 12, *n* = 297), iron binding (cluster 14, 225) etc., were represented by a single cluster. However, several clusters represented more than one functional classification. For instance, clusters 13 (*n* = 113), 15 (*n* = 356), 18 (*n* = 109), and 20 (*n* = 138) exhibit a broader level of functional activities related to protein modification, transferase activity, catalytic activity, and metabolism. Overall, this analysis suggests that many pathways respond to virus infection in a modular fashion by co-expressed gene components. Also, identification of co-expressed genes within the same cluster indicates possible interactions between different pathways responding to virus-induced stress.

### Transcription factors involved in response to BCMV infection

The roles of different transcription factors (TFs) under various biotic and abiotic stress conditions have been extensively studied [28, 55, 57, 58]. To identify the level and pattern of expression of different TFs, we extracted the HMM motifs of known legume transcription factor (TF) families available in the legume transcription factor database (http://legumetfdb.psc.riken.jp/) and identified the matched regions in DE gene sequences (e-value < 10^−10^ and 90 % identity). This database includes sixty-one TF families from *Glycine max*, *Lotus japonicus*, and *Medicago truncatula*. The TFs were categorized using separate datasets for upregulated genes and downregulated genes for each virus treatment (Tables 5 and 6). A total of thirty-three and twenty-one TF families were detected in NLI and BCMV-S2 treatments, respectively (Table 6, Additional file 23: Table S5). These families represented a total of 246 (5.26 % of total DE genes) and 101 (4.81 % of total DE genes) differentially expressed TFs in the NL1-I and the BCMV-S2 treatment, respectively (Table 6, Additional file 23: Table S5). Seven TF classes were most abundant in the DE gene dataset. These classes were (R1)R2R3_Myb, AP2_EREBP, bHLH, C2H2_Zn, Myb_related, NAC, and WRKY_Zn (Table 6). The members of different TF families had varying contribution in upregulated and downregulated datasets. For example, NAC and WRKY_Zn families had more members with induced expression (Table 6). In contrast, the Myb_related and bHLH families have more members that were repressed (Table 6). Overall, the analysis indicated that diverse TF classes showed significant but varying responses under the two virus infections.

**Table 5:** Number of differentially expressed transcription factors (TFs) on NL1-I and BCMV-S2 virus infections

	NL1-I	BCMV-S2
Number of Detected TF Families	33	21
Number of Detected TFs	246 (5.26 %)	101 (4.81 %)
Upregulated	138 (2.95 %)	56 (2.67 %)
Downregulated	108 (2.31 %)	45 (2.14 %)

Significant DE genes (padj < 0.05) were used to identify putative TFs for individual virus treatment. Hidden Markov Motifs (HMM) representing 61 different legume transcription factor families [44] were compared against the DE genes and putative TFs were detected using 90 % sequence homology and e-value ≤ 10^−10^

**Table 6:** Most abundant transcription factor (TF) families detected in DE dataset for NL1-I and BCMV-S2 infections

	NL1-I		BCMV-S2	
Most Abundant	Induced	Repressed	Induced	Repressed
(R1)R2R3_Myb	11	10	5	5
AP2_EREBP	12	2	4	3
bHLH	9	17	7	8
C2H2_Zn	6	8	1	1
Myb_related	3	13	1	6
NAC	20	2	7	1
WRKY_Zn	25	6	11	1

The numbers of genes corresponding to each TF family were counted after blast results. Datasets for upregulated and downregulated genes for each virus treatment was used separately

## Discussion

This study describes the regulatory landscape of *P. vulgaris* and the associated expression changes at two different stages of BCMV infection. We further described the identification and genome sequence of a previously unknown BCMV strain used in this study. At the start of experiment, the identity of one BCMV strain was not known. We utilized the unmapped sequences to produce a *de novo* assembly of the genomes of two virus strains used in this study. Comparison of genome sequences from the two viruses with known virus sequences in NCBI (http://www.ncbi.nlm.nih.gov/) revealed them as most similar to the strain NL1 of BCMV. However, further sequence comparison of both strains suggests the two viruses are distinct strains of BCMV. To clearly differentiate the transcriptional response to two BCMV strains and to further characterize the severity of symptoms of BCMV-S2, we conducted RNAseq on two time points during infection on a universally susceptible line, stringless green refugee. The analysis of alternate splicing (AS) and differential gene expression (DGE) patterns clearly indicate a strain-specific host response upon infection. A larger number of significant (padj < 0.05) AS events were observed for the BCMV-S2 (342) strain than the NL1-I (172) strain. However, the trend was opposite in terms of DGE, and fewer genes showed differential expression in BCMV-S2 (2099) than for the NL1-I (4676) strain. Overall, these results indicate that the viruses represent two different strains of BCMV which activate differential host transcriptional responses upon infection and that these differences may correlate both to the expanded host range of S2 and the increased severity of the symptoms in susceptible cultivars. Moreover, there is also a high level of overlap between DE genes (upregulated and downregulated) across virus treatments – which suggests that common regulatory pathways respond to the different virus stimuli. In contrast, identification of uniquely expressed genes implies strain-specific induction in each case.

Plants respond to stress conditions in part by producing spliced isoforms [27, 48, 50] and by changing transcriptional expression levels [59–61]. Thus, we performed a detailed genome wide characterization of host transcriptional response at 4 and 8 days after BCMV inoculation of *P. vulgaris* L. These stages were selected to represent represented early onset of infection to full systemic spread of virus infection in common bean leaves as determined by ELISA. We determined that although we did not observe symptoms until day 12, we could identify viral reads as early as day 4. We identified a number of putative AS events, previously unannotated in the common bean genome. The distribution ratios of detected AS events were consistent across healthy and virus inoculated samples. However, the frequency of occurrence was different for various AS events. Intron retention (IR) and alternate acceptor events represent the most frequent types of AS events (31–35 %). This observation is consistent with previous studies in other monocot and dicot species [27, 62]. An increase in systemic virus stress over time increases the number of significant AS events. These results were consistent with previous observation of increases in AS events after *Panicum mosaic virus* infection in *Brachypodium distachyon* [27]. However, we also identified few significant AS forms with relative reduction in number after virus infection. Similar observations were noticed in some other stress related studies [50–52] The decreased number of some AS events under severe virus infection suggests the possible role of mRNA degradation machinery to eliminate most of the unproductive transcripts [63, 64]. Statistical analysis revealed a number of significant AS events with comparatively more spliced transcripts in the BCMV-S2 than the NL1-I virus treatment, which clearly shows distinct host responses upon infection using different virus strains. Although the data in this study is limited by the nature of sequencing reads and will require further experiments for validation, these results do provide preliminary evidence about modulation of AS landscape during virus infections. Future studies aimed at putative splicing motifs and underlying variants in AS regulators can provide further insights into molecular mechanisms underlying host-virus interactions. For example, previous analysis of AS during virus infection identified multiple termination codons in the splicing regulators [27], which can potentially be targeted for decay by cellular degradation machinery to keep unproductive transcripts under check [63–65].

Similarly, host gene expression levels show drastic changes for a large number of genes after virus infection. Also, the expression changes were clearly different for NL1-I and BCMV-S2 strains, which further highlights the differential host responses upon infection with two strains of the same virus. In our study, the initial sampling stage (day 4) defines the start of host response as revealed by expression changes in only 1 and 2 genes for NL1-I and BCMV-S2 treatments, respectively. In particular, a noticeable repression in expression level of a ribulose bisphosphate carboxylase gene (Phvul.004G073400) was detected under both virus treatments. This observation suggests that photosynthetic pathways quickly respond under pathogen stress conditions. This result is consistent with previous data during infection of *Arabidopsis thaliana* with Turnip mosaic virus (TuMV) [66]. Analysis of transcript abundance patterns during systemic virus infection stage (day 8) displayed more evident expression shifts in the host transcriptome machinery. Most of the DE genes exhibit induced expression level in NL1-I (61.4 %) and BCMV-S2 (71.9 %) treatments, while fewer genes were downregulated (38.6 % in NL1-I and 28.1 % in BCMV-S2). The majority of upregulated genes involved Leucine-Rich repeat receptor-like protein kinases, calcium-binding EF-hand family members, pathogenesis-related (PR) proteins, oxidative stress related genes, and WRKY transcription factors. Many of these gene classes have a known role during stress response and pathogen resistance [67–71]. Pathway analysis also identified specific gene classes related to kinase activity, receptor activity, cell death, protein modification, protein metabolism, ribonucleoprotein complex, ribosome, cellular metabolic activity, gene expression and translation were particularly induced during virus stress. Also, similar pathways were activated upon NL1-I and BCMV-S2 infections, which suggest the concerted reprogramming of major defense-related modules involved in diverse stress responses. Similar analysis using down-regulated genes identified that pathways associated with photosynthetic, metabolic process, carbohydrate metabolism, transferase activity, and catalytic activity were repressed under virus stress. It would be interesting to analyze other time points, perhaps six days, ten days or even twelve days to determine how the viral response progressed over time. This may provide a more accurate account of changes in transcript abundance due to differences in viral accumulation observed during the analysis of the reads from four and eight days with S2 having approximately eight times the reads compared to NL1 at day two.

Corresponding changes in photosynthetic pathways and various metabolic activities after pathogen infection have been reported earlier [72–74]. These results indicate that cells extensively modulate the metabolic activity and energy production during virus stress conditions. Switching-on defense mechanism and respiratory processes is a cost-intensive process [74–76], which might occur at the expense of photosynthesis turn off [74]. Reduced photosynthetic gene activity might have been associated with chlorosis or decreased green tissue surface area after systemic virus spread. Overall, this tight metabolic regulation is critical for resistance response or for specific defense mechanisms during virus infection [70]. Specific models explaining the concerted regulation of metabolic activity and photosynthesis have been suggested [77, 78]. These models propose a system level coordination between various cellular and metabolic processes to overcome pathogen infection. Our results from co-expressed gene modules representing various response pathways support previous models.

Various transcription factors (TFs) have well-characterized roles under different stress conditions [55, 57, 58]. Thus, we assessed the complexity of host-virus interactions by identifying TF families activated or repressed during virus stress. Approximately 4.8 % to 5.3 % differentially expressed genes were putative TFs with both up-regulated (2.67 % to 2.95 % for BCMV-S2 and NL1-I, respectively) and down-regulated (2.14 % to 2.31 % for BCMV-S2 and NL1-I, respectively) expression patterns under virus stress. Similar induction of six OsNAC transcription factors has been reported as a result of *Rice stripe virus* and *Rice tungro spherical virus* infection [79]. Nonetheless, several TF families identified in this study have known role in response to pathogen and other stresses [80–83]. This can provide further opportunities for exploring their underlying molecular mechanisms in the regulation of different aspects of host-virus interactions.

## Conclusions

Infection of two different strains of bean common mosaic virus (BCMV) clearly showed differential transcriptome response in the common bean (*Phaseolus vulgaris* L.) plants. Plants undergo genome level changes in transcript expression levels and patterns during virus infection. Systemic virus infection rewires gene regulatory networks in the host plant. Our analyses increase the understanding of system level changes associated with BCMV infection, and provide a basis for future explorations of plant response to infection with BCMV.

## Additional files

Additional file 1: Figure S1. Sequncing analysis workflow for virus genome identification, aletrnate splicing (AS) analysis and differential gene expression analysis. Analysis was perfomed using different software tools suitable for each part. (JPG 608 kb) Additional file 2: Figure S4. Distribution of normalized viral reads across the viral genome. Reads were aligned to the viral genomes and normalized to determine ratio of reads corresponding to gene size and plotted. Each region of the genome is represented by the corresponding gene name. Error bars indicate the standard error across three replicates. (JPG 397 kb) Additional file 3: Table S1. Reads obtained for each sample from individual sequencing lane of Illumina Hi-Seq platform. (DOC 28 kb) Additional file 4: Table S2. Statistics of raw read cleaning and mapping for each sequenced sample. (DOC 51 kb) Additional file 5: Table S3. Proportion of Virus reads to the total transcriptome reads per treatment. (DOC 28 kb) Additional file 6: Variants from BCMVS2 reads on the BCMVS2 genome assembly. Variant-call-format file, showing single-nucleotide variants within the BCMVS2 genome. (ZIP 3 kb) Additional file 7: Variants from BCMVS2 reads on the NL1I genome assembly. Variant-call-format file, showing single-nucleotide variants between the BCMVS2 genomic reads and the NL1I genome assembly. (ZIP 13 kb) Additional file 8: Variants from NL1I reads on the BCMVS2 genome assembly. Variant-call-format file, showing single-nucleotide variants between the NL1I genomic reads and the BCMVS2 genome assembly. (ZIP 12 kb) Additional file 9: Variants from NL1I reads on the NL1I genome assembly. Variant-call-format file, showing single-nucleotide variants within the NL1I genome. (ZIP 3 kb) Additional file 10: Figure S2. Various AS events identifed in the annotated common bean (Phaseolus vulgaris L.) genome [33]. Alternate Splicing Transcriptional Landscape Visualization Tool, ASTALAVISTA, [38] was used to visualize the nature and distribution pattern of various splicing events. (JPG 43 kb) Additional file 11: Alternate splice events from the bean genome assembly. (ZIP 82 kb) Additional file 12: Figure S3. Distribution pattern of various alternate splicing (AS) events observed in healthy and virus inoculated samples at 4 and 8 days after infection. Each plot represents the samples as follows: (A) Day4 Healthy, (B) Day4 NL1-I, (C) Day4 BCMV-S2, (D) Day8 Healthy, (E) Day8 NL1-I, and (F) Day8 BCMV-S2. The transcripts were constructed using cufflinks v2 [36] and low abundance transcripts (FPKM < 0.3) were eliminated for splicing detection. Various AS events were categorized using Alternate Splicing Transcriptional Landscape Visualization Tool, ASTALAVISTA, [38]. (JPG 566 kb) Additional file 13: Significant alternate-splice transcripts for NL1-I at 4 days after infection. (ZIP 3 kb) Additional file 14: Significant alternate-splice transcripts for BCMV-S2 at 4 days after infection. (ZIP 4 kb) Additional file 15: Significant alternate-splice transcripts for NL1-I at 8 days after infection. (ZIP 20 kb) Additional file 16 Significant alternate-splice transcripts for BCMV-S2 at 8 days after infection. (ZIP 37 kb) Additional file 17: Table S4. Genes expressed at different read count criteria in healthy and virus inoculated samples at 4 and 8 dpi. (DOC 73 kb) Additional file 18: Differentially-expressed genes for NL1-I at 8 days after infection. (ZIP 139 kb) Additional file 19: Differentially-expressed genes for BCMV-S2 at 8 days after infection. (ZIP 66 kb) Additional file 20: Gene ontology terms for differentially expressed genes under NL1-I infection. (ZIP 43 kb) Additional file 21: Gene ontology terms for differentially expressed genes under BCMV-S2 infection. (ZIP 41 kb) Additional file 22: Genes in clusters determined by normalized expression values from the DE genes in each virus treatment. (ZIP 20 kb) Additional file 23: Table S5. Transcription factor (TF) families detected in differential expression datasets with gene count in each family. (DOC 73 kb)
