# Supplementary figures and images for: Dynamic transcriptome profiling of Bean Common Mosaic Virus (BCMV) infection in Common Bean (Phaseolus vulgaris L.)
Source: BMC Genomics. 2016 Aug 11;17:613. doi: 10.1186/s12864-016-2976-8 (PMC4982238; doi:10.1186/s12864-016-2976-8)

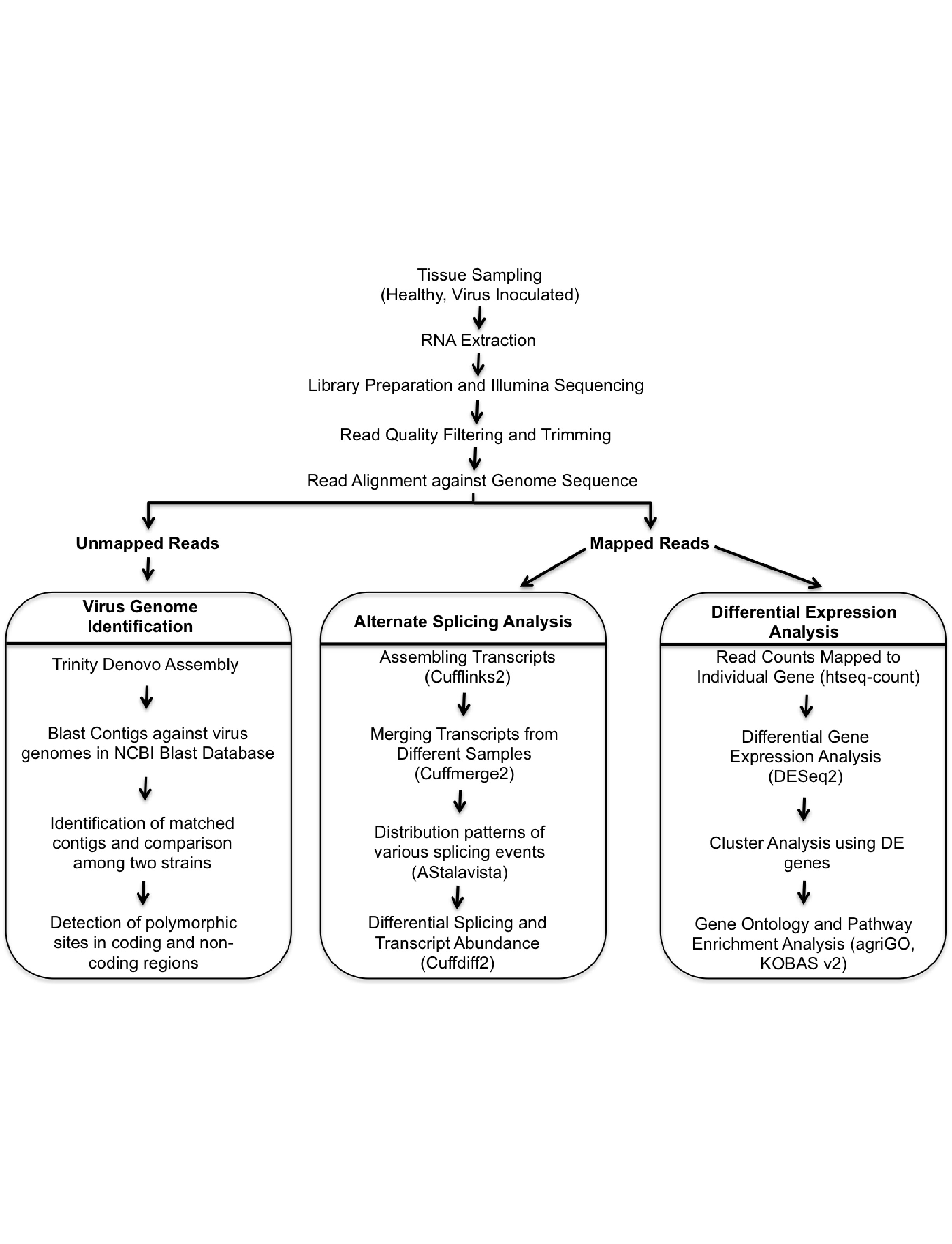

Supplement: Additional file 1: Figure S1. — Sequncing analysis workflow for virus genome identification, aletrnate splicing (AS) analysis and differential gene expression analysis. Analysis was perfomed using different software tools suitable for each part. (JPG 608 kb) [file 12864_2016_2976_MOESM1_ESM.jpg]

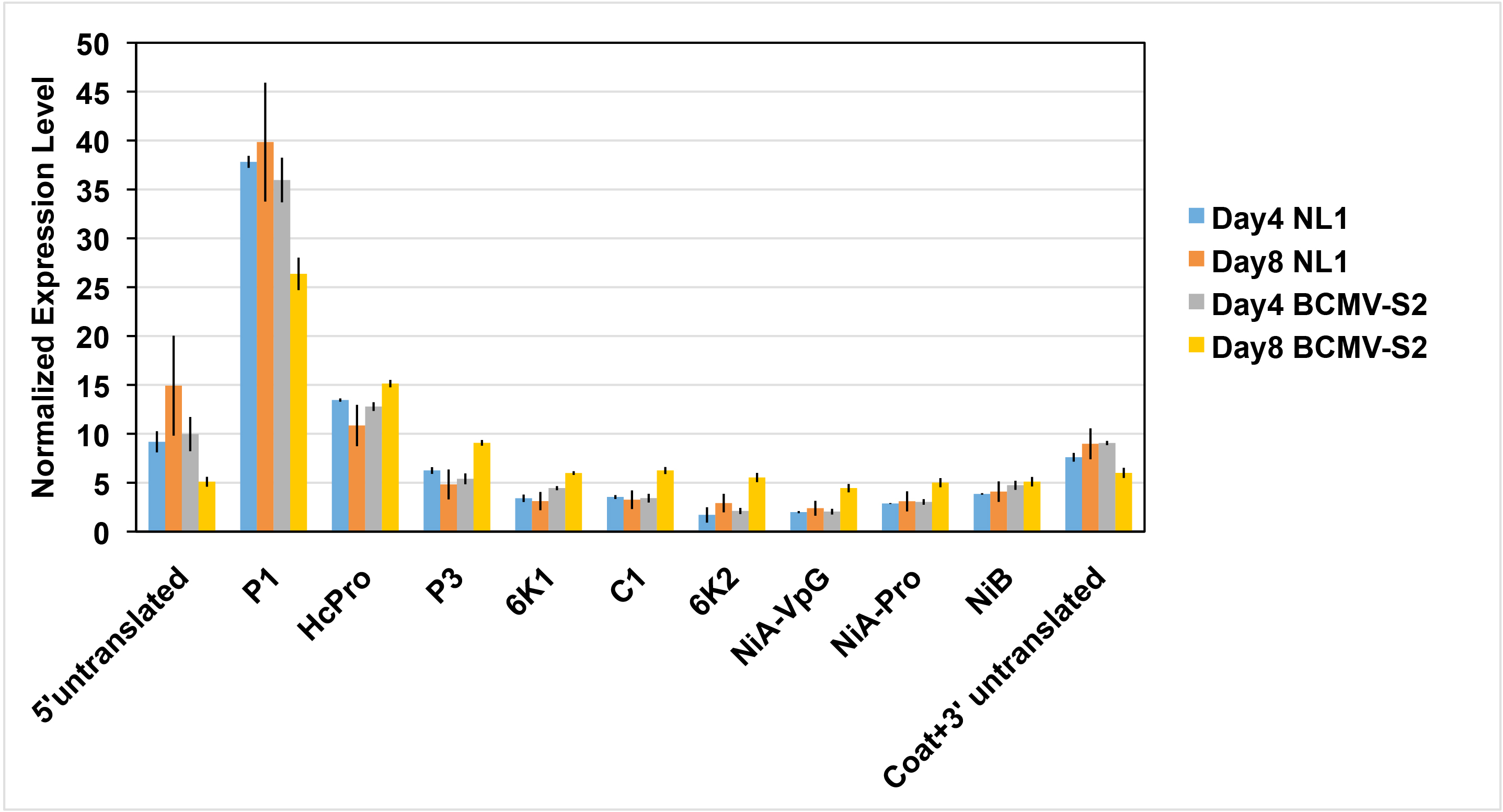

Supplement: Additional file 2: Figure S4. — Distribution of normalized viral reads across the viral genome. Reads were aligned to the viral genomes and normalized to determine ratio of reads corresponding to gene size and plotted. Each region of the genome is represented by the corresponding gene name. Error bars indicate the standard error across three replicates. (JPG 397 kb) [file 12864_2016_2976_MOESM2_ESM.jpg]

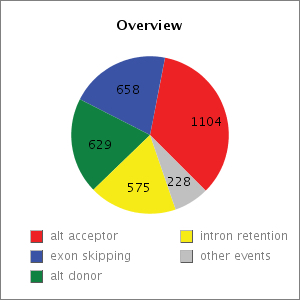

Supplement: Additional file 10: Figure S2. — Various AS events identifed in the annotated common bean (Phaseolus vulgaris L.) genome [33]. Alternate Splicing Transcriptional Landscape Visualization Tool, ASTALAVISTA, [38] was used to visualize the nature and distribution pattern of various splicing events. (JPG 43 kb) [file 12864_2016_2976_MOESM10_ESM.jpg]

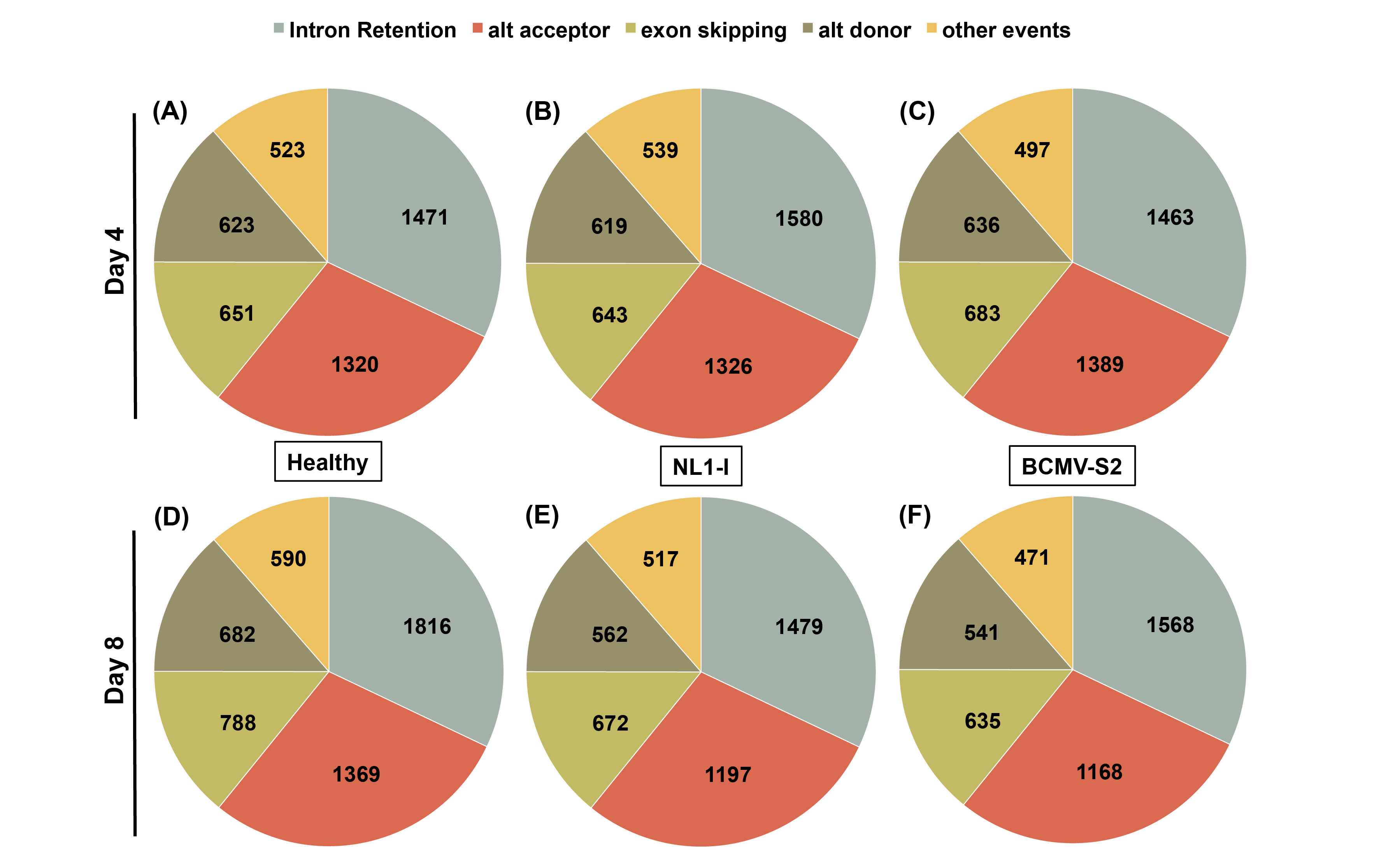

Supplement: Additional file 12: Figure S3. — Distribution pattern of various alternate splicing (AS) events observed in healthy and virus inoculated samples at 4 and 8 days after infection. Each plot represents the samples as follows: (A) Day4 Healthy, (B) Day4 NL1-I, (C) Day4 BCMV-S2, (D) Day8 Healthy, (E) Day8 NL1-I, and (F) Day8 BCMV-S2. The transcripts were constructed using cufflinks v2 [36] and low abundance transcripts (FPKM < 0.3) were eliminated for splicing detection. Various AS events were categorized using Alternate Splicing Transcriptional Landscape Visualization Tool, ASTALAVISTA, [38]. (JPG 566 kb) [file 12864_2016_2976_MOESM12_ESM.jpg]
