# Supplementary material for: Dynamic transcriptome profiling of Bean Common Mosaic Virus (BCMV) infection in Common Bean (Phaseolus vulgaris L.)
Source: BMC Genomics. 2016 Aug 11;17:613. doi: 10.1186/s12864-016-2976-8 (PMC4982238; doi:10.1186/s12864-016-2976-8)
Supplement: Additional file 3: Table S1. — Reads obtained for each sample from individual sequencing lane of Illumina Hi-Seq platform. (DOC 28 kb) [file 12864_2016_2976_MOESM3_ESM.doc]

**Table S1. Reads obtained for each sample from individual sequencing lane of Illumina Hi-Seq platform**.

| **Time** | **Treatment** | **Lane1** | **Lane2** | **Lane3** |
| --- | --- | --- | --- | --- |
| Day4 | Healthy | 33870250 | 26869453 | 36872141 |
|  | BCMV-S2 | 33712224 | 35253793 | 34002739 |
|  | NL1-I Strain | 32801754 | 33699188 | 30897015 |
| Day8 | Healthy | 26714003 | 31453830 | 26957980 |
|  | BCMV-S2 | 32273085 | 35520376 | 38677848 |
|  | NL1-I Strain | 26593895 | 33851936 | 32230217 |
