# Supplementary material for: Dynamic transcriptome profiling of Bean Common Mosaic Virus (BCMV) infection in Common Bean (Phaseolus vulgaris L.)
Source: BMC Genomics. 2016 Aug 11;17:613. doi: 10.1186/s12864-016-2976-8 (PMC4982238; doi:10.1186/s12864-016-2976-8)
Supplement: Additional file 4: Table S2. — Statistics of raw read cleaning and mapping for each sequenced sample. (DOC 51 kb) [file 12864_2016_2976_MOESM4_ESM.doc]

**Table S2. Statistics of raw read cleaning and mapping for each sequenced sample**.

|  | **Input Reads** | **Cleaned Reads** | **Percentage of Cleaned Reads** | **Dropped Reads** | **Percentage of Dropped Reads** | **Mapped** | **Unique Mapped** | **Multi Position Match** | **Unmapped** |
| --- | --- | --- | --- | --- | --- | --- | --- | --- | --- |
| D4HR1 | 33870250 | 33830710 | 99.88% | 39540 | 0.12% | 33830484 | 27504531 | 6325953 | 226 |
| D4HR2 | 33712224 | 33674960 | 99.89% | 37264 | 0.11% | 33674706 | 28510552 | 5164154 | 254 |
| D4HR3 | 32801754 | 32766612 | 99.89% | 35142 | 0.11% | 32766065 | 26299403 | 6466662 | 547 |
| D4NL1-IR1 | 26714003 | 26684800 | 99.89% | 29203 | 0.11% | 26684559 | 22579724 | 4104835 | 241 |
| D4NL1-IR2 | 32273085 | 32238148 | 99.89% | 34937 | 0.11% | 32237842 | 27373442 | 4864400 | 306 |
| D4NL1-IR3 | 26593895 | 26565384 | 99.89% | 28511 | 0.11% | 26564577 | 21662975 | 4901602 | 807 |
| D4BCMV-S2R1 | 26869453 | 26836864 | 99.88% | 32589 | 0.12% | 26836635 | 22788385 | 4048250 | 229 |
| D4BCMV-S2R2 | 35253793 | 35207916 | 99.87% | 45877 | 0.13% | 35207670 | 28079840 | 7127830 | 246 |
| D4BCMV-S2R3 | 33699188 | 33656170 | 99.87% | 43018 | 0.13% | 33655706 | 27944358 | 5711348 | 464 |
| D8HR1 | 31453830 | 31414222 | 99.87% | 39608 | 0.13% | 31413923 | 25239904 | 6174019 | 299 |
| D8HR2 | 35520376 | 35476556 | 99.88% | 43820 | 0.12% | 35476377 | 29008216 | 6468161 | 179 |
| D8HR3 | 33851936 | 33808072 | 99.87% | 43864 | 0.13% | 33807772 | 28189472 | 5618300 | 300 |
| D8NL1-IR1 | 36872141 | 36825541 | 99.87% | 46600 | 0.13% | 36825130 | 28979778 | 7845352 | 411 |
| D8NL1-IR2 | 34002739 | 33961970 | 99.88% | 40769 | 0.12% | 33960471 | 27053521 | 6906950 | 1499 |
| D8NL1-IR3 | 30897015 | 30857974 | 99.87% | 39041 | 0.13% | 30857666 | 24909342 | 5948324 | 308 |
| D8BCMV-S2R1 | 26957980 | 26924064 | 99.87% | 33916 | 0.13% | 26922723 | 20655739 | 6266984 | 1341 |
| D8BCMV-S2R2 | 38677848 | 38628645 | 99.87% | 49203 | 0.13% | 38626671 | 27988414 | 10638257 | 1974 |
| D8BCMV-S2R3 | 32230217 | 32189959 | 99.88% | 40258 | 0.12% | 32188829 | 24433094 | 7755735 | 1130 |
| **Sum** | **582251727** | **581548567** | **17.9781** | **703160** | **0.0219** | **581537806** | **469200690** | **112337116** | **10761** |
| **Average** | **32347318.1** | **32308253.7** | **0.998** | **39064.4** | **0.0426** | **1129245128** | **910896849** | **218348279** | **21296** |
| **Median** | **33250471** | **33211391** | **0.9988** | **39574** | **0.0841** | **2224815550** | **1793283146** | **431532404** | **42338** |
| **Std Dev** | **3573556.3** | **3568219.8** | **8.5E-05** | **5865.3** | **0.1671** | **4416865035** | **3560266889** | **856598146** | **84129** |

Here, D4H (Day4 Healthy), D4NL1-I (Day4 NL1-I), D4BCMV-S2 (Day4 BCMV-S2), D8H (Day8 Healthy), D8NL1-I (Day8 NL1-I), D8BCMV-S2 (Day8 BCMV-S2). The raw reads were cleaned using Trimmomatic [32] and high quality reads were mapped against *P. vulgaris* genome (V1) using Tophat software [34].
