# Supplementary material for: Dynamic transcriptome profiling of Bean Common Mosaic Virus (BCMV) infection in Common Bean (Phaseolus vulgaris L.)
Source: BMC Genomics. 2016 Aug 11;17:613. doi: 10.1186/s12864-016-2976-8 (PMC4982238; doi:10.1186/s12864-016-2976-8)
Supplement: Additional file 5: Table S3. — Proportion of Virus reads to the total transcriptome reads per treatment. (DOC 28 kb) [file 12864_2016_2976_MOESM5_ESM.doc]

**Table S3. Proportion of Virus reads to the total transcriptome reads per treatment.**

|  | **Virus Read count** | **Transcriptome reads** | **% viral reads/ sample transcriptome** |
| --- | --- | --- | --- |
| D4HR | 63 | 100272282 | 6.28289E-05 |
| D4NL1-S2 | 11870 | 85488332 | 0.013884936 |
| D4NL1-Iowa | 96239 | 95700950 | 0.10056222 |
| D8HR | 212 | 100698850 | 0.000210529 |
| D8NL1-S2 | 1568510 | 101645485 | 1.543118221 |
| D8NL1-Iowa | 2330959 | 97742668 | 2.384791665 |
