# Supplementary material for: Dynamic transcriptome profiling of Bean Common Mosaic Virus (BCMV) infection in Common Bean (Phaseolus vulgaris L.)
Source: BMC Genomics. 2016 Aug 11;17:613. doi: 10.1186/s12864-016-2976-8 (PMC4982238; doi:10.1186/s12864-016-2976-8)
Supplement: Additional file 17: Table S4. — Genes expressed at different read count criteria in healthy and virus inoculated samples at 4 and 8 dpi. (DOC 73 kb) [file 12864_2016_2976_MOESM17_ESM.doc]

**Table S4. Genes expressed at different read count criteria in healthy and virus inoculated samples at 4 and 8 dpi**.

|  |  | Genes Detected | | |
| --- | --- | --- | --- | --- |
| **Time** | **Treatment** | **Read count ≥ 5** | **Read count ≥ 3** | **Read count ≥ 1** |
| Day4 | Healthy | 21071 (77.80%) | 21634 (79.88%) | 22880 (84.48%) |
|  | BCMV-S2 | 20829 (76.91%) | 21484 (79.33%) | 22759 (84.04%) |
|  | NL1-I | 21007 (77.56%) | 21634 (79.88%) | 22862 (84.42%) |
| Day8 | Healthy | 21058 (77.76%) | 21656 (79.96%) | 22824 (84.28%) |
|  | BCMV-S2 | 21313 (78.70%) | 21888 (80.82%) | 22962 (84.79%) |
|  | NL1-I | 21077 (77.83%) | 21648 (79.94%) | 22624 (83.54%) |

The quality filtered reads were aligned to available *P. vulgaris* (version 1) genome using bowtie2 aligner in Tophat [36]. The unique reads aligned to each annotated *P. vulgaris* gene model was counted using Htseq-count [39] python script.
