# Supplementary material for: Dynamic transcriptome profiling of Bean Common Mosaic Virus (BCMV) infection in Common Bean (Phaseolus vulgaris L.)
Source: BMC Genomics. 2016 Aug 11;17:613. doi: 10.1186/s12864-016-2976-8 (PMC4982238; doi:10.1186/s12864-016-2976-8)
Supplement: Additional file 23: Table S5. — Transcription factor (TF) families detected in differential expression datasets with gene count in each family. (DOC 73 kb) [file 12864_2016_2976_MOESM23_ESM.doc]

**Table S5. Transcription factor (TF) families detected in differential expression datasets with gene count in each family**.

| **NL1-I** | | | | **BCMV-S2** | | | |
| --- | --- | --- | --- | --- | --- | --- | --- |
| **Induced** | **TFs** | **Repressed** | **TFs** | **Induced** | **TFs** | **Repressed** | **TFs** |
| (R1)R2R3_Myb | 11 | (R1)R2R3_Myb | 10 | (R1)R2R3_Myb | 5 | (R1)R2R3_Myb | 5 |
| ABI3VP1 | 2 | ABI3VP1 | 4 | ABI3VP1 | 1 | ABI3VP1 | 1 |
| AP2_EREBP | 12 | AP2_EREBP | 2 | AP2_EREBP | 4 | AP2_EREBP | 3 |
| *na* | *na* | ARF | 1 | bHLH | 7 | bHLH | 8 |
| *na* | *na* | atypical_MYB | 1 | bZIP | 3 | *na* | *na* |
| bHLH | 9 | bHLH | 17 | C2C2_Zn-Dof | 1 | *na* | *na* |
| bZIP | 5 | bZIP | 2 | *na* | *na* | C2C2_Zn-GATA | 1 |
| C2C2_Zn-CO-like | 4 | C2C2_Zn-GATA | 4 | C2H2_Zn | 1 | C2H2_Zn | 1 |
| C2H2_Zn | 6 | C2H2_Zn | 8 | C3H-TypeI | 1 | *na* | *na* |
| C3H-TypeI | 2 | C3H-TypeI | 3 | CCAAT_HAP2 | 2 | *na* | *na* |
| CAMTA | 3 | *na* | 1 | *na* | *na* | GARP_ARRB | 1 |
| CCAAT_HAP2 | 3 | CCAAT_HAP2 | *na* | GARP_G2-like | 2 | GARP_G2-like | 1 |
| CPP | 1 | CPP | 1 | HB | 3 | HB | 7 |
| *na* | *na* | GARP_ARRB | 1 | JUMONJI | 1 | *na* | *na* |
| GARP_G2-like | 4 | GARP_G2-like | 1 | *na* | *na* | HSF | 2 |
| GRAS | 2 | GRAS | 2 | MADS | 1 | MADS | 2 |
| HB | 8 | HB | 7 | Myb_related | 1 | Myb_related | 6 |
| HMG-box | 1 | *na* | *na* | NAC | 7 | NAC | 1 |
| HSF | 1 | *na* | *na* | PHD | 3 | PHD | 3 |
| JUMONJI | 1 | JUMONJI | 1 | SBP | 2 | SBP | 2 |
| *na* | *na* | LIM | 1 | WRKY_Zn | 11 | WRKY_Zn | 1 |
| MADS | 2 | MADS | 2 |  |  |  |  |
| Myb_related | 3 | Myb_related | 13 |  |  |  |  |
| NAC | 20 | NAC | 2 |  |  |  |  |
| *na* | *na* | Nin-like | 1 |  |  |  |  |
| PHD | 5 | PHD | 8 |  |  |  |  |
| S1Fa-like | 1 | *na* | *na* |  |  |  |  |
| SBP | 2 | SBP | 4 |  |  |  |  |
| *na* | *na* | TCP | 2 |  |  |  |  |
| Trihelix | 3 | Trihelix | 1 |  |  |  |  |
| TUB | 2 | *na* | *na* |  |  |  |  |
| WRKY_Zn | 25 | WRKY_Zn | 6 |  |  |  |  |
| *na* | *na* | zf-HD | 2 |  |  |  |  |

The putative TFs in differential expression datasets were identified by blast match with known HMM motifs from *Medicago truncatula*, *Lotus japonicus*, and *Glycine max*.
